# Targeting β-Cell Plasticity: A Promising Approach for Diabetes Treatment

**DOI:** 10.3390/cimb46070453

**Published:** 2024-07-18

**Authors:** Esmaeel Ghasemi Gojani, Sweta Rai, Farzaneh Norouzkhani, Salma Shujat, Bo Wang, Dongping Li, Olga Kovalchuk, Igor Kovalchuk

**Affiliations:** Department of Biological Sciences, University of Lethbridge, Lethbridge, AB T1K 3M4, Canada; esmaeel.ghasemigojan@uleth.ca (E.G.G.);

**Keywords:** β-cells, dedifferentiation, transdifferentiation, neogenesis, regeneration

## Abstract

The β-cells within the pancreas play a pivotal role in insulin production and secretion, responding to fluctuations in blood glucose levels. However, factors like obesity, dietary habits, and prolonged insulin resistance can compromise β-cell function, contributing to the development of Type 2 Diabetes (T2D). A critical aspect of this dysfunction involves β-cell dedifferentiation and transdifferentiation, wherein these cells lose their specialized characteristics and adopt different identities, notably transitioning towards progenitor or other pancreatic cell types like α-cells. This process significantly contributes to β-cell malfunction and the progression of T2D, often surpassing the impact of outright β-cell loss. Alterations in the expressions of specific genes and transcription factors unique to β-cells, along with epigenetic modifications and environmental factors such as inflammation, oxidative stress, and mitochondrial dysfunction, underpin the occurrence of β-cell dedifferentiation and the onset of T2D. Recent research underscores the potential therapeutic value for targeting β-cell dedifferentiation to manage T2D effectively. In this review, we aim to dissect the intricate mechanisms governing β-cell dedifferentiation and explore the therapeutic avenues stemming from these insights.

## 1. Introduction

Globally, around 422 million people have Diabetes Mellitus (DM or diabetes), with most living in low- and middle-income countries. Diabetes causes 1.5 million deaths annually worldwide. In the United States, approximately 37.3 million people, or about 11% of the population, have diabetes, with Type 2 Diabetes (T2D) accounting for 90–95% of cases. Projections indicate that the global number of adults with diabetes will rise to 643 million by 2030 and 783 million by 2045. 

**Figure 1:**
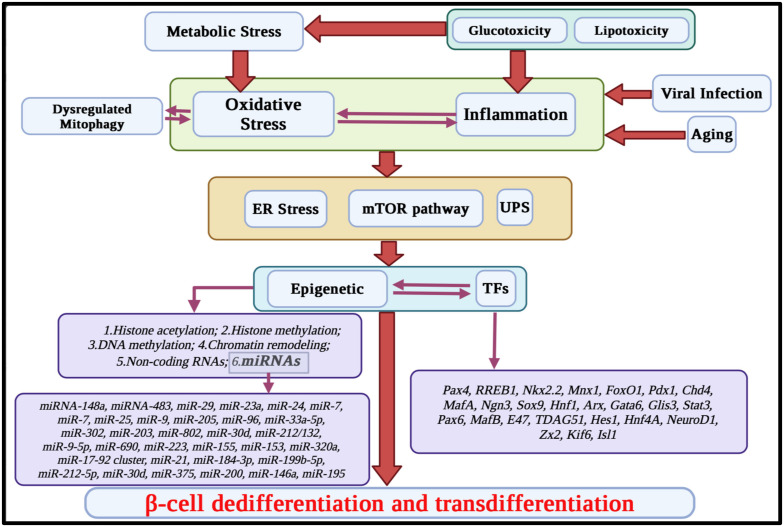
The mechanism of β-cell dedifferentiation and transdifferentiation. The process of β-cell dedifferentiation and transdifferentiation involves metabolic stresses such as glucotoxicity, lipotoxicity, viral infections, and aging. These stresses induce oxidative stress and inflammation, disrupting pathways such as ER stress, the mTOR pathway, and the UPS. This disruption affects the activities of transcription factors and epigenetic factors, including various miRNAs, leading to β-cell dedifferentiation and transdifferentiation. Abbreviations: endoplasmic reticulum: ER; ubiquitin–proteasome system: UPS; transcription factors: TFs. The information included in this figure is based on the research findings cited in this review paper.

Findings indicate that changing β-cell plasticity plays an important role in the incidence of all three main forms of diabetes (see Box 1). In T2D, for example, β-cell dedifferentiation and transdifferentiation are more critical than β-cell loss [1,2]. Various factors, including metabolic stress, inflammation [3], and viral infection [4], contribute to these processes. They exert their effects through pathways such as the mTOR pathway, ER stress, and by altering the transcriptional activity of β-cells, targeting key transcription and epigenetic factors (Figure 1).

The concept of β-cell dedifferentiation was introduced by Gershengorn et al. in 2004 after they observed that β-cells in human pancreatic islets vanished and were replaced by fibroblast-like precursor cells. They also noted that these cells could redifferentiate into α- and β-cells under certain growth conditions. This concept was further confirmed by the findings of Dor et al. (2007), Accili et al. (2012), Wang et al. (2014), and Cinti et al. (2016) [5]. β-cell dedifferentiation is characterized by the appearance of progenitor-like biomarkers, such as Ngn3, Oct4, Aldh1a3, Nanog, L-Myc, and Sox9, and the disappearance or downregulation of β-cell-enriched biomarkers, including *MafA*, *Ins*, *Nkx6.1*, *Glut2*, and *Pdx-1*. This shift results in β-cells losing their functionality, which can ultimately lead to the incidence of diabetes [5,6]. Similar to rodents, β-cell dedifferentiation occurs in human pancreatic islets. However, unlike in rodents, *Ngn3* is not upregulated in human pancreatic islets during dedifferentiation. *Aldh1a3*, on the other hand, is upregulated in both rodents and humans [7]. The transdifferentiation of β-cells into other pancreatic cell types, such as α- and δ-cells, is another form of β-cell plasticity that occurs in both rodents and humans, much like dedifferentiation [6]. The transdifferentiation of β-cells into α-cells is typically marked by the loss of β-cell differentiation markers and the acquisition of α-cell biomarkers, such as glucagon and the Arx transcription factor [8]. The transdifferentiation of pancreatic endocrine cells, including α- and δ-cells, into β-cells is observed alongside reports of pancreatic exocrine cells, like acinar cells, also transdifferentiating into β-cells. This suggests a novel approach for treating diabetes [9].

The dedifferentiation and transdifferentiation of β-cells are observed across all diabetes types. In Type I Diabetes (T1D), inflammatory reactions are pivotal in triggering these processes, inducing ER stress and modifying gene expression and protein synthesis in β-cells [10]. In T2D, metabolic stresses linked to obesity, insulin resistance, and high levels of glucose and lipids similarly contribute to β-cell dedifferentiation and transdifferentiation [11]. 

Mitigating and reversing β-cell dedifferentiation and transdifferentiation offer promising treatments for all three main types of diabetes. Various approaches, including natural products, dietary interventions, and common antidiabetic therapies, can help mitigate β-cell dedifferentiation and transdifferentiation.

In addition, in vitro β-cell neogenesis followed by the transplantation of newly differentiated β-cells is a promising method for treating all types of diabetes, particularly T1D. We have highlighted new findings in this area.

β-cell regeneration and proliferation represent another strategy for treating all types of diabetes. Suppressing Dual-Specificity Tyrosine-Regulated Kinase 1A (DYRK1A) [12], antagonizing glucagon receptors [13,14,15,16], and inhibiting polyamine biosynthesis [17] are potential treatments to promote β-cell regeneration and proliferation.

Box 1 **Definitions.**

 β-cell plasticity refers to the ability of β-cells to alter their identity and function in response to specific conditions. This trait is not exclusive to β-cells; other pancreatic cells, such as α-cells, also exhibit this adaptability. The primary aspects of β-cell plasticity are β-cell dedifferentiation and transdifferentiation [18].

 β-cell dedifferentiation is triggered by chronic metabolic stresses like hyperglycemia, endoplasmic reticulum (ER) stress, and inflammatory cytokines. This process involves mature β-cells losing their differentiated characteristics and reverting to a progenitor-like or undifferentiated state [2].

 β-cell transdifferentiation involves the conversion of mature β-cells into other islet cell types, such as α-cells, under certain conditions.

 β-cell regeneration is the process of restoring or increasing the number of functional pancreatic β-cells, which produce insulin. This process can occur through the body’s natural mechanisms, involving two main pathways:

 1—β-cell proliferation: Existing β-cells divide to increase their numbers.

 2—Neogenesis from progenitor cells: Pancreatic stem or progenitor cells differentiate into new β-cells [19].

 T1D—Type 1 Diabetes: an autoimmune condition where the immune system attacks insulin-producing β-cells in the pancreas, leading to an inability to produce insulin. It accounts for about 10% of diabetes cases and is usually diagnosed in childhood or adolescence, though it can occur at any age [20].

 T2D—Type 2 Diabetes: the most common form caused by insulin resistance and relative insulin deficiency. It is often associated with obesity, a sedentary lifestyle, and genetic factors. While it is typically diagnosed in adulthood, it is increasingly seen in children and teens due to rising obesity rates. Management can involve lifestyle changes, medications, or insulin [21,22].

 GDM—Gestational Diabetes Mellitus arises when the body struggles to produce sufficient insulin to counteract the insulin resistance induced by placental hormones such as estrogen, cortisol, and human placental lactogen [23].

In this review, we explore the mechanisms behind β-cell dedifferentiation and transdifferentiation and provide insights into the possibility of mitigating these processes by identifying new therapeutic targets, medications, and treatments. Additionally, we highlight recent discoveries related to β-cell neogenesis and regeneration as potential treatments for all three types of diabetes.

## 2. The Main Mechanisms Involved in β-Cell Function and Identity

### 2.1. Inflammation

Inflammation, whether originating from the β-cells themselves or from resident macrophages in the pancreas, plays a critical role in the function and maintenance of β-cells, thereby contributing to the development of diabetes [3]. Pro-inflammatory cytokines like IL-1β, IL-6, and TNFα induce β-cell dedifferentiation in cultured human cells, human islets, and mouse islets, contributing to β-cell dysfunction in T2D. IL-1β is the most potent among them, downregulating *Forkhead Box O1* (*FoxO1*), a key transcription factor for maintaining β-cell identity. Additionally, treatment with proinflammatory cytokines was associated with a translocation of FoxO1 to the nucleus, the loss of nuclear *Nkx6.1* expression, and the emergence of *Aldh1a3*, indicating β-cell dedifferentiation [24]. Accordingly, in vivo, anti-inflammatory treatments with anti-IL-1β, anti-TNFα, or NF-kB inhibiting sodium salicylate improved insulin secretion from isolated islets. While anti-TNFα therapy partially prevented the loss of β-cell identity gene expression, the combined antagonism of IL-1β and TNFα produced a synergistic effect in restoring insulin secretion. Although inflammation plays a significant role in β-cell dedifferentiation in vitro, its impact on in vivo insulin secretion improvement appears to be more complex, with additional factors potentially involved [25]. 

The pro-inflammatory factor BMP-2, upregulated in the islets of diabetic individuals, contributes to the inhibition of β-cell function and proliferation. BMP-2 disrupts β-cell maturity through epigenetic changes and decreased NeuroD1 activity, contributing to β-cell dysfunction in diabetes. Exposure to BMP-2 for 10 days impaired glucose-stimulated insulin secretion (GSIS) and β-cell proliferation, correlating with decreased expressions of key β-cell genes like *Ins1*, *Ucn3*, and *Ki67*. BMP-2-induced β-cell dysfunction was also linked to an increased expression of dedifferentiation regulators (*Id1-4*, *Hes-1*, and *Hey-1*), a reduction in H3K27 acetylation, and decreased NeuroD1 DNA binding activity [26].

TGF-β, a versatile cytokine, coordinates various cellular processes such as growth, proliferation, differentiation, apoptosis, and immune responses. It plays a crucial role in regulating β-cell dedifferentiation, preserving β-cell function, and managing β-cell mass [27]. Inhibitors targeting TGF-β receptor I (TGFβ1) have shown potential in restoring β-cell identity by influencing the expression of β-cell-specific transcription factors under severe diabetic conditions [28].

Patients with chronic pancreatitis (CP) are at risk of developing diabetes due to the gradual loss of functional β-cells. In a mouse model for CP-related diabetes (CPRD), researchers found that β-cell loss is driven by TGFβ1-induced epithelial–mesenchymal transition (EMT), not apoptosis. They also observed increased angiogenesis in CP patients’ inflamed pancreata and in mice subjected to pancreatic duct ligation, a surgical procedure used in research to induce pancreatitis. However, when angiogenesis was blocked with an anti-vascular endothelial growth factor receptor 2 antibody (DC101), the EMT process reverted to β-cell apoptosis. This indicates that angiogenesis helps β-cells survive in the inflamed pancreas, suggesting a potential therapeutic target for CPRD [29].

Activin is a protein that belongs to the TGF-β superfamily. It plays crucial roles in various biological processes, including embryonic development, cell proliferation, differentiation, and tissue homeostasis. Its signaling influences the balance between α- and β-cell populations, impacting glucose homeostasis. Accordingly, it has been found that Activin can suppress genes in α-cells such as *Arx4* and upregulate β-cell genes such as *Pax4*, which accelerates the process of transdifferentiation from α- to β-cells [30]. 

In diabetes, stromal cell-derived factor-1 (SDF-1), a chemokine, is implicated in the dedifferentiation of islet β-cells. Elevated SDF-1 levels correlate with the increased dedifferentiation of β-cells in pancreatic tissues. Experimental inhibition of SDF-1 led to a higher proportion of dedifferentiated cells, while overexpression reduced their numbers. Despite the diabetes-induced upregulation of *SDF-1*, its activity may decline, thereby impacting dedifferentiation. SDF-1 exerts its effects through CXCR4, activating AKT and FoxO1 [31].

T2D exhibits hyperglycemia and inflammation, paralleled by the upregulation of prostaglandin E2 (PGE2). A systemic blockade of the PGE2 receptor EP3 promotes β-cell proliferation and mass without affecting α-cells. EP3 blockade reverses islet gene expression changes in diabetic mice, restoring normal islet architecture. Additionally, it increases *Glp1r* expression and activates Nrf2, potentially protecting β-cells from oxidative stress. EP3 blockade shows promise for enhancing β-cell mass in T2D [32].

Insulitis, or inflammation of β-cells, is most commonly associated with T1D. Under this condition, β-cells are usually exposed to a variety of cytokines, including IFNγ, IL-1β, TNFα, and IL-17, all of which are able to trigger ER stress and β-cell dedifferentiation [10]. There is some evidence indicating that the direct interaction of autoimmune T cells with β-cells may be responsible, but not sufficient, for changes in the β-cell identity in T1D patients [33]. However, the islet microenvironment in T1D has been reported to generate CD4 T cells that target proinsulin, suggesting that proinflammatory mediators from islets may cause β-cell dedifferentiation in individuals with T1D [34]. Moreover, it has been found that exposing β-cells to double-stranded RNA, mimicking a viral infection that could trigger T1D, reduces the expressions of β-cell-enriched genes like *MafA* and *Ins* while increasing progenitor markers. This effect is driven by NF-kB within the β-cells and the interferon alpha released by neighboring cells [35,36]. 

The ablation of β-cells, which is associated with T1D, induces the transdifferentiation of α-cells into β-cells [37]. This indicates that cell plasticity could be used as a potential treatment for T1D. It is worth mentioning that in the context of T1D, the immune system does not attack α-cells [38].

### 2.2. Metabolic Stress

Metabolic stressors, including glucotoxicity, lipotoxicity, and glucolipotoxicity, have been demonstrated to impair β-cell function and identity by disrupting key metabolic pathways. 

Glucotoxicity has been shown to inhibit β-cell regeneration. In a mouse model, where β-cells are ablated, the usual regenerative response includes β-cell proliferation and the normalization of blood glucose levels. However, if the β-cell loss leads to severe hyperglycemia (above 28 mmol/L), regeneration fails, causing permanent diabetes, despite some surviving β-cells entering the cell cycle. Reducing hyperglycemia with insulin, SGLT2 inhibitors, or a ketogenic diet for about three weeks can partially restore β-cell mass and aid in diabetes recovery, indicating that extreme glucotoxicity hinders regeneration. Gene expression analysis suggests that high glucose levels cause metabolic stress and suppress key cell-cycle genes, potentially blocking regeneration [39].

Persistent hyperglycemia maintains a cycle of β-cell dysfunction. Using advanced optical imaging, researchers found that streptozotocin (STZ) predominantly damages larger islets in the pancreatic core, leading to a significant reduction in β-cell mass. However, even in hyperglycemic STZ-treated mice, a considerable number of β-cells remain but with a decreased expression of *Glut2* (*Slc2a2*), indicating impaired β-cell maturity. Islet gene expression studies confirm this downregulation and suggest that hyperglycemia causes β-cell dysfunction more than ablation. Islet transplantation partially restored some markers of islet function but not the overall β-cell mass [40].

The hyperactive isoform of carbohydrate response-element-binding protein (ChREBPβ) plays a crucial role in the response of β-cells to hyperglycemic stress. It is involved in adaptive β-cell expansion when faced with metabolic challenges like prolonged glucose exposure and HFD. However, chronic overexpression of *ChREBPβ* in β-cells can lead to a loss of β-cell identity, increased apoptosis, reduced β-cell mass, and diabetes. Deleting ChREBPβ can prevent β-cell “glucolipotoxicity”, while the overexpression of *ChREBPα* or activation of the antioxidant Nrf2 pathway can mitigate ChREBPβ-induced β-cell death [41].

Stearoyl CoA desaturase (SCD), an enzyme pivotal in lipid metabolism, is highly expressed in β-cells and human islets. The silencing of SCD prompts inflammation, ER stress, and elevated *IAPP* mRNA levels. Conversely, treatment with SCD products counteracts these effects. Moreover, when SCD is knocked down and exposed to palmitate, dedifferentiation markers are induced. Notably, SCD knockdown alone disrupts β-cell identity, diminishing mature β-cell markers and compromising insulin secretion [42].

Accordingly, it has been found that the inhibition of stearoyl-CoA desaturase 1 (SCD1) is associated with increased methylation in *Pdx1* and *MafA* promoter regions. This inhibition also skews pancreas development towards α-cell formation, disrupting the α-cell-to-β-cell ratio and islet structure. Consequently, islet dysfunction occurs, characterized by impaired insulin secretion and elevated glucagon levels [43].

Oxidative stress originating from hyperglycemia and hyperlipidemia can cause a significant decline in human pancreatic β-cell function. This decline is characterized by the activation of ER stress and the nuclear translocation of FoxO1. This stress response results in the downregulation of the maturity genes *MafA* and *Pdx1* along with the upregulation of the progenitor markers *Sox9* and *Hes1*, suggesting a potential process of partial dedifferentiation. These findings highlight the critical importance of targeting antioxidant mechanisms to preserve functional β-cell mass, particularly in the early stages of T1D [44].

During the insulin resistance phase of T2D, β-cells exhibit persistently high calcium levels. Initially, this adaptation may assist β-cells in managing the metabolic stress induced by hyperglycemia. However, prolonged exposure to elevated calcium levels may lead to alterations in β-cell function and identity, as well as diabetic complications. Single-cell RNA sequencing of pancreatic islets from prediabetic and diabetic mice unveiled a diabetes-specific transcriptomic profile. It highlighted *Anxa10*, a gene linked to prediabetes, as being upregulated by persistently high calcium levels in β-cells. Pseudotime analysis (computational ordering of cells in the progression of differentiation) suggested that disturbances in calcium influx during the insulin resistance phase and heightened Anxa10 expression could induce mitochondrial dysfunction, ER stress, and β-cell transdifferentiation into acinar cells, offering new insights into diabetes progression [45].

PARP1 is related to metabolic stress through its involvement in DNA repair mechanisms and cellular responses to stressors such as oxidative stress and inflammation. During metabolic stress, such as high glucose levels or lipid overload, PARP1 can be activated excessively, leading to the increased production of poly(ADP-ribose) polymers (PARylation). Research shows that *PARP1* knockout (KO) mice exhibit resistance to diabetes, and inhibiting PARP1, particularly with PJ34, alleviates complications in type 2 diabetes patients. Complete PARP1 depletion halted β-cell formation, whereas its inhibition enhanced β-cell differentiation and maturation via p38 MAPK phosphorylation and Ngn3 reactivation, implying PARP1’s necessity for proper β-cell development [46].

Maintaining lysosomal activity and mitophagy is crucial for the differentiation and maturation of β-cells. The Niemann-Pick C1 protein (NPC1) on the lysosome membrane is essential for intracellular cholesterol transport and lysosome function. Research links NPC1 mutations to pancreatic islet dysfunction in T2D. NPC1 depletion in mice impairs β-cell viability, expansion, and GSIS, disrupting mitochondrial function through compromised lysosomal activity and mitophagy, indicating metabolic stress [47].

Insulin granule maturation in pancreatic β-cells relies heavily on phosphatidylinositol transfer protein α (PITPNA). Reduced PITPNA levels in β-cells from individuals with T2D result in impaired GSIS, diminished β-cell mass, and increased ER stress, reflecting metabolic stress. The conditional deletion of *PITPNA* in mice causes hyperglycemia and compromised insulin processing. Silencing *PITPNA* in human islets yields similar defects that can be reversed by restoring PITPNA levels [48]. 

### 2.3. Age-Related Dynamics of β-Cell Dedifferentiation and Transdifferentiation

The impact of age on β-cell dedifferentiation and transdifferentiation is increasingly recognized, with studies revealing a significant rise in dedifferentiated cells among older age groups. This phenomenon is accompanied by diminished FoxO1 activity and reduced *UCN3* expression in aging β-cells along with a decline in adaptive unfolded protein response (UPR) components. Glucose-regulated protein 94 (GRP94), also known as heat shock protein 90B1 (HSP90B1), is an ER chaperone that plays a crucial role in protein folding, quality control, and the maintenance of ER homeostasis. Lowering GRP94 levels has been shown to induce dedifferentiation, while restoring GRP94 levels preserves β-cell identity in aging cells [49].

β-cell regeneration becomes challenging with advancing age, particularly in the context of adult-onset diabetes. In middle-aged rats, β-cell aging is notably influenced by compromised duct cell plasticity and β-cell dedifferentiation. Despite observed increases in hypertrophy and hyperplasia, the regenerative potential of functional β-cell mass is limited. This implies the critical role of β-cell dedifferentiation and potential transdifferentiation in the aging process [50].

Excessive glycolysis may contribute to age-related β-cell dysfunction and the loss of cellular identity. Both aged and diabetic mouse β-cells exhibit common dysfunctional traits, including heightened glucose sensitivity and compromised cellular integrity. They also share an increase in glycolysis driven by elevated *Nmnat2* expression, which is crucial for NAD synthesis. Knocking out *Got1*, a gene associated with glycolysis, replicated these traits, which are observed in aging and diabetes. Restoring the β-cell function and integrity was achieved by modulating glycolysis or the Nmnat2 activity in *Got1* KO β-cells [51].

### 2.4. Sex-Dependent Variations in Pancreatic Islet Plasticity

Sex influences β-cell plasticity, as evidenced by the differences observed in pancreatic islet structures between male and female mice. Male control mice exhibit larger pancreatic islets and experience a greater reduction in islet and β-cell areas after STZ exposure compared to females. Conversely, female mice treated with STZ show lower rates of β-cell apoptosis. Hydrocortisone (HC) administration results in a higher proportion of α-cells in female islets, which is accompanied by a slight increase in β-cell apoptosis. Healthy female mice demonstrate a higher rate of α-to-β-cell transdifferentiation, which decreases with HC treatment, while STZ treatment increases α-cell transdifferentiation to β-cells in males but not females. These findings underscore the impact of sex hormones on islet morphology and function [52].

### 2.5. The Impacts of Chemicals on β-Cell Plasticity

Compounds like ethanol, STZ, glucocorticoids, and environmental pollutants impact different aspects of diabetes, including the dedifferentiation and transdifferentiation of β-cells.

Alcohol addiction is a serious global issue, responsible for 5.9% of all deaths worldwide. Regular alcohol consumption heightens the risk of metabolic diseases, including T2DM, compared to nonconsumption. Chronic alcohol consumption results in reduced insulin levels, β-cell apoptosis, and decreased β-cell mass along with upregulated *Fibroblast Growth Factor 21* (*FGF21*) and its receptor mRNA levels, indicating FGF21 resistance. Short-term ethanol exposure does not immediately impair β-cell function, but prolonged exposure progresses to significant dysfunction. Chronic binge drinking is linked to reductions in insulin, the insulin-to-glucagon ratio, the GSIS response, and NKX2.2 and PDX1 levels, highlighting alcohol’s impact on β-cell dedifferentiation [53]. Additionally, there is a negative correlation between alcohol consumption and blood adiponectin levels [54], which diminish insulin expression and secretion [55] as well as β-cell survival [56].

STZ is a naturally occurring compound used to induce diabetes in experimental models by selectively destroying insulin-producing β-cells in the pancreas through DNA alkylation and oxidative stress. It targets β-cells specifically due to their high expression of the *Glut2* glucose transporter, which facilitates STZ uptake and leads to cell death. However, some β-cells with lower *Glut2* expression survive the initial damage but experience long-term functional and phenotypic changes, remaining in a low-Glut2 state. It has been found that some interventions, such as islet transplantation and glucose normalization, can partially restore β-cell function and *Glut2* expression [57,58]. STZ treatment potentially promotes β-cell regeneration and transdifferentiation in pancreatic islets. This includes the emergence of vimentin+/MafB+ cells, which can differentiate into insulin-producing β-like cells, and the possibility of α-cells transdifferentiating into β-cells under specific conditions [1,59]. 

Glucocorticoids such as dexamethasone and hydrocortisone can induce β-cell dedifferentiation through various mechanisms, including triggering ER stress, changes in gene expression [60], impaired β-cell glucose metabolism, and reduced sensitivity to glucose stimulation, leading to altered insulin secretion patterns and decreased GSIS [61,62]. Furthermore, excess glucocorticoid exposure during fetal development can permanently alter β-cell mass and function, resulting in long-term consequences for glucose homeostasis [63].

Bisphenols (BPs) and phthalates are classes of industrial chemicals widely used in the production of plastics and other consumer products. These chemicals have been linked to higher diabetes rates, particularly affecting β-cells. Exposure to these chemicals disrupts electrical activity, mitochondrial function, gene expression, and β-cell function, leading to altered GSIS and impaired β-cell proliferation [64]. Studies show mixed results, with some indicating heightened insulin secretion and content, while others suggest diminished GSIS or insulin levels. These substances can also impact β-cell survival, with higher doses or prolonged exposure often leading to increased cell death [65]. BPs induce ER stress, mitochondrial dysfunction, and oxidative stress in β-cells, contributing to cellular damage, apoptosis, and plasticity [66,67]. The effects on β-cell proliferation vary, with some studies showing increased proliferation at lower doses [68] and others observing reduced growth at higher concentrations [69]. Bisphenol A (BPA) acts as an endocrine disruptor, altering gene expression in β-cells during development and producing long-term metabolic disturbances. Early-life exposure to BPA increases β-cell mass and proliferation through estrogen receptor β (ERβ), resulting in hyperinsulinemia that precedes insulin resistance [70,71].

Phthalates affect insulin secretion in β-cells variably depending on the duration and concentration of exposure. Acute high-concentration exposure increases basal insulin secretion (BIS) without affecting GSIS. In contrast, prolonged exposure typically inhibits both BIS and GSIS, indicating impaired insulin synthesis and β-cell function, often coupled with reduced β-cell survival and increased cellular stress [64,72].

## 3. Molecular Pathways Involved in β-Cell Plasticity

### 3.1. ER Stress

ER stress results from the buildup of misfolded proteins, activating the UPR to restore normalcy. Environmental factors like inflammation and oxidative stress can trigger ER stress, which is implicated in T1D and T2D onset [73,74]. ER stress activates the UPR, which is mediated by sensors like IRE1α, PERK, and ATF6, which aim to restore ER homeostasis [75]. 

Prolonged ER stress can lead to cellular dysfunction and apoptosis, particularly affecting pancreatic β-cells. When β-cells experience ER stress, they need to activate mechanisms to adapt and manage this stress effectively. Chronic stress induces transcriptional and translational changes, impairing β-cell function and identity regulators. Despite having some capacity to adapt to stress, β-cells under prolonged stress can eventually lose their functional identity. This means they no longer produce insulin efficiently, which is crucial for regulating blood sugar levels.

Single-cell RNA sequencing from T1D patients reveals severe disruptions in stress adaptation, indicating a role for β-cell adaptive exhaustion in diabetes progression [76].

A study by Hugo Lee et al. (2020) discovered that the β-cell deletion of IRE1α in Non-Obese Diabetic (NOD) mice, in the T1D model, led to transient β-cell dedifferentiation, temporarily protecting against immune-mediated β-cell destruction. Additionally, IRE1α influences the stability of *TXNIP* mRNA, impacting NLRP3 inflammasome activation and cell death pathways [77].

The X-box binding protein 1 (XBP1) serves as a pivotal regulator of the UPR within the ER. Extensive evidence implies the crucial role of XBP1 in preserving β-cell identity and function, especially during periods of metabolic stress. The depletion of XBP1 results in β-cell dysfunction, dedifferentiation, and heightened vulnerability to diabetes, underscoring its significance in maintaining β-cell homeostasis and regulating insulin secretion [78].

Like T1D, T2D is also characterized by increased ER stress and altered insulin processing, leading to a loss of β-cell identity and function. The transition from impaired glucose tolerance to diabetes, as a hallmark of T2D, is marked by a significant increase in ER stress markers and a decrease in β-cell phenotype markers. β-cell dedifferentiation appears as the disease progresses, with islets showing reduced expressions of key genes such as *Nkx6.1* and *MafA*. Additionally, these changes were accompanied by an increase in altered proinsulin processing and intracellular localization, indicating a deterioration of the β-cell secretory machinery [73].

ATF4, a key regulator of ER stress responses, contributes to pancreatic β-cell dysfunction in T2D. It induces phosphodiesterase 4D (*PDE4D*) expression, disrupting cAMP signaling and accelerating T2D progression. Pharmacological inhibition of the ATF4 pathway reduces *PDE4D* expression, improving glucose tolerance and insulin secretion. The administration of Sephin1, a selective inhibitor of a holophosphatase with a modulatory impact on ER stress, enhances glucose metabolism in Akita mice by increasing insulin-positive areas and *ATF4* expression. Conversely, β-cell-specific *ATF4* knockout (βAtf4-KO) mice exhibit exacerbated diabetes, with increased dedifferentiation markers and a reduced expression of the Atonal homolog 8 transcription factor (*Atoh8*) [79,80].

ATF4 is also a key player in integrated stress response (ISR). ISR is a complex cellular signaling pathway in eukaryotic cells that manages and mitigates various stress stimuli. Essential for maintaining cellular homeostasis, the ISR can be triggered by intrinsic and extrinsic factors such as hypoxia, amino acid deprivation, glucose deprivation, viral infections, and oxidative stress [81]. The ISR is driven by the phosphorylation of eukaryotic translation initiation factor 2 alpha (eIF2α) by one of four kinases: PERK, GCN2, PKR, or HRI. This phosphorylation reduces global protein synthesis, conserving resources and alleviating the load on the protein-folding machinery. At the same time, it promotes the translation of specific genes, such as the transcription factor ATF4, which, in turn, aids in cellular adaptation to stress by upregulating the genes involved in both protein folding and amino acid metabolism [82]. The activation of the ISR in β-cells is found to regulate oxidative stress, a key player in β-cell dedifferentiation. This supports the observations indicating that the phosphorylation of eIF2α has a protective impact on β-cells against oxidative stress caused by insulin resistance through an ATF4-dependent mechanism [83]. Amino acid deficiency, another inducer of the ISR, stimulates the binding of uncharged tRNA to GCN2, leading to the autophosphorylation of GCN2 and subsequent phosphorylation of eIF2α [84]. This phosphorylation of eIF2α is associated with an increase in ATF4 levels, which upregulates the genes involved in the synthesis and metabolism of amino acids [85]. The upregulation of ATF4 also increases the expression of *sesterin2*, thereby inhibiting mTORC1. It is noteworthy that the chronic activation of mTORC1 during the insulin resistance phase negatively impacts β-cell mass and proliferation [86].

In summary, ATF4 upregulation, whether through ER stress or through the ISR, has a great impact on the β-cell fate. 

Both high-glucose- and cytokine-induced ER stress activate intracellular calcium and reactive oxygen species (ROS) signals in pancreatic β-cells. These signals trigger calcineurin (CN)/NFATc2 and PI3K/AKT pathways, regulating gene expression that is critical for β-cell function. While inducing the endocrine differentiation gene *RFX6*, they suppress “disallowed” genes like *MCT1*, which are detrimental to β-cell identity. CN/NFATc2, along with co-activator p300 and co-repressor HDAC1, orchestrates these gene expression changes. Prolonged stress or disrupted CN/NFATc2 signaling leads to β-cell dedifferentiation, marked by reduced *RFX6* and elevated *MCT1* levels [87].

### 3.2. Angiotensin Receptors

Angiotensin receptors are cell surface receptors that bind and respond to angiotensin peptides produced by the renin–angiotensin system (RAS). There is an interaction between the renin–angiotensin aldosterone system (RAAS) and ER stress in pancreatic β-cells, particularly in the context of T2D [88]. The activation of the Angiotensin II (Ang II) receptor is linked to the initiation of β-cell dedifferentiation and the development of T2D. This occurs through the activation of the RAS and subsequent NFκB activation [89].

The activation of Angiotensin II exerts its negative impact on β-cell plasticity by increasing ER stress markers and reducing insulin secretion in clonal INS-1E β-cells and human islets. Accordingly, it has been found that the inhibition of Ang II production or the blockade of its receptors alleviates ER stress and restores insulin secretion. These findings elucidate a potential mechanism linking pancreatic RAAS activation to β-cell dysfunction in T2D, highlighting the therapeutic potential of RAAS inhibitors in T2D management [88].

### 3.3. mTOR Pathway

The protein kinase mTOR (the mechanistic target of rapamycin) regulates cell growth, proliferation, and survival, impacting cellular metabolism and protein synthesis. mTOR activity decreases in mice fed a high-fat diet (HFD), leading to the initial enlargement but subsequent diminishment of pancreatic islets. This decrease in mTOR activity correlates with increased β-cell death, reduced levels of myeloid cell leukemia 1 (Mcl-1), a Bcl-2 family member crucial for pancreatic β-cell survival, and impaired insulin secretion in response to glucose. Additionally, the activation of AMPK by palmitic acid (PA) degrades Mcl-1 via pathways such as ERK and GSK3β, further contributing to β-cell dysfunction and loss of identity [90].

The growth factor receptor-bound protein 10 (GRB10), a negative regulator of insulin and mTORC1 signaling, is significantly elevated in the islets of diabetic mice and insulinoma cells treated with high glucose. β-cell-specific knockout of *GRB10* in mice increased β-cell mass and function along with enhanced mTORC1 signaling and reduced β-cell dedifferentiation, reversible by rapamycin. Conversely, the overexpression of *GRB10* in MIN6 cells induced β-cell dedifferentiation, indicating potential therapeutic targeting of GRB10 to address β-cell dysfunction in diabetes [91].

The deletion of the β-cell-specific gene *Raptor*, a component of mTORC1, resulted in decreased β-cell mass, the adoption of α-cell characteristics, and loss of β-cell identity, even after glucose normalization. A *Raptor* deficiency directly impaired β-cell identity, mitochondrial function, and protein synthesis, leading to β-cell dysfunction. Additionally, *Raptor* loss activated *MafB*, a transcription factor associated with α-cells, and genes specific to α-cells, promoting a transition from β- to α-cell identity. These findings highlight mTORC1’s role in maintaining β-cell identity and suppressing α-cell characteristics under normal glucose conditions [92].

Additionally, the mTORC1 pathway regulates β-cell functional maturation through epigenetic mechanisms. Utilizing microarray, MeDIP-seq, and ATAC-seq analyses, it has been uncovered that DNMT3A-dependent DNA methylation and PRC2-dependent H3K27me3 modification are key silencing mechanisms in immature β-cells lacking *Raptor.* Overexpressing *DNMT3A* partially reverses the immature transcriptome pattern and restores impaired GSIS in *Raptor*-deficient β-cells. Furthermore, Raptor directly regulates *PRC2/EED* and *H3K27me3* expressions, suggesting a complex interplay between nutrient sensing, epigenetic modifications, and β-cell function, with implications for T2D [93].

Rictor, a key component of mTORC2, regulates β-cell proliferation and function, with long chain acyl-CoA synthetase 4 (Acsl4) potentially downstream. *Acsl4* is positively regulated by Rictor at both transcriptional and post-translational levels in mouse β-cells. Adenovirus-mediated *Acsl4* expression rescued β-cell dysplasia in βRicKO islets but not dysfunction. However, *Acsl4* could not fully restore glucose oxidation in *Rictor*-deficient β-cells, instead promoting lipid oxidation. Epigenetic analyses revealed signatures of β-cell dedifferentiation and an altered oxidative response in *Rictor*-lacking cells expressing *Acsl4*, suggesting a context-dependent role for *Acsl4* in β-cell dedifferentiation under reduced Rictor/mTORC2 activity [94].

mTORC1 activation under chronic metabolic stress leads to increased levels of the phosphatases PHLPP1 and PHLPP2 in diabetic β-cells. These elevated phosphatases contribute to β-cell failure in diabetes by regulating survival kinases such as AKT and MST1. Inhibiting PHLPPs genetically enhances β-cell survival and function in diabetic models and human T2D islets, suggesting a potential therapeutic target for diabetes [95].

### 3.4. Role of Ubiquitin–Proteasome System in the Pancreatic β-Cell Plasticity

The ubiquitin–proteasome system (UPS) regulates cellular protein degradation, impacting various biological processes like inflammation and apoptosis. Emerging evidence suggests its crucial involvement in pancreatic β-cell transdifferentiation in diabetes, presenting a potential treatment avenue [96,97].

The UPS, which includes E3 ligases and deubiquitinases (DUBs), regulates several aspects of β-cell function. The inhibition of deubiquitinase USP1, a key player in β-cell dedifferentiation, either through genetic intervention or using the small-molecule inhibitor ML323, restored the epithelial phenotype of β-cells. Conversely, the overexpression of USP1 induced dedifferentiation, which was mediated by modulating the expression of the inhibitor of differentiation 2 (ID2). In an STZ-induced mouse model of β-cell dedifferentiation, treatment with ML323 improved hyperglycemia [98].

Ubiquitin-Specific Peptidase 7 (USP7) stabilizes Ngn3 by deubiquitination. The conditional knockout of Usp7 in mice reduces islet formation and causes hyperglycemia due to impaired Ngn3-mediated endocrine specification. Pharmacological inhibition of USP7 in human iPSC models also decreases *Ngn3* progenitors and impairs β-cell differentiation, highlighting the importance of the USP7-Ngn3 axis in preventing β-cell dedifferentiation [99].

Ubiquitin-mediated degradation of *Ngn3*, regulated by PRMT1-mediated arginine 65 methylation, is essential for β-cell differentiation. *Protein arginine methyltransferase-1* (*PRMT1*) knockout in human embryonic stem cells (hESCs) leads to Ngn3 accumulation in the cytoplasm, reducing its transcriptional activity and preventing endocrine cell differentiation. This methylation acts as a molecular switch, ensuring Ngn3’s proper localization and function, thus preventing β-cell dedifferentiation [100]. 

## 4. Transcriptional Regulation in β-Cell Plasticity

Transcription factors are crucial for establishing and maintaining the mature phenotype of β-cells as well as regulating their plasticity through processes such as dedifferentiation, transdifferentiation, and regeneration. Key transcription factors, including Pdx1, Ngn3, Pax4, MafA, FoxO1, and Nkx6.1, are essential for β-cell identity, function, and survival. The dysregulation of these factors can result in dedifferentiation, where β-cells lose their mature state and revert to a progenitor-like condition. 

Furthermore, alterations in the transcriptional network can drive the transdifferentiation of β-cells into other islet cell types, such as α- or δ-cells. Modulating transcription factors like Pdx1, Ngn3, and MafA shows potential for reprogramming other cell types into insulin-producing β-like cells, thereby facilitating β-cell regeneration [101]. The information on key transcription factors involved in dedifferentiation (De), transdifferentiation (Tra), neogenesis (Neo), and proliferation (Pro) is summarized in Table 1.

The table is divided into seven columns: “Transcription Factors” lists the names of the transcription factors involved in β-cell dedifferentiation, transdifferentiation, neogenesis, or proliferation (De/Tra/Neo/Pro). “Full Name” provides the full names of these transcription factors. “Target Genes” describes the genes regulated by the transcription factors. “Impact on Genes” explains how each transcription factor affects the target genes (induction (I) or repression (R)). “De/Tra/Neo/Pro” indicates how the transcription factors impact β-cell plasticity and regeneration. “Impact on β-Cell Plasticity/Regeneration” describes whether the transcription factors have a positive (P) or negative (N) effect on β-cell plasticity/regeneration. “References” lists the sources used for each transcription factor’s explanation. Abbreviations: P: Positive, N: Negative, NM: Not Mentioned, I: induction, R: Repression.

## 5. Translational Control

The eukaryotic initiation factor 5A (eIF5A), crucial for β-cell identity and function, undergoes a post-translational modification by deoxyhypusine synthase (DHPS) to activate its translational activity. In mice lacking DHPS, there is a significant reduction in the proteins essential for β-cell identity, leading to rapid-onset diabetes during β-cell maturation. Further investigation revealed that eIF5A regulates the translation of key genes like *Ins1*, *Glut2*, *Ucn3*, and *Chga* during β-cell maturation, underscoring the pathway’s significance in β-cell function maintenance. Targeting mRNA translation in β-cells, particularly by modulating the eIF5A pathway, could present a therapeutic approach to correcting diabetes-related defects [189]. 

## 6. Epigenetic-Related Targets

### 6.1. Histon Modifications

Histone modifications act as epigenetic regulators that control the expression of key β-cell genes. The disruption of these histone marks contributes to β-cell dedifferentiation, while resetting them is crucial for transdifferentiation approaches aimed at generating functional β-like cells [190].

### 6.2. Histone Acetylation

Sirtuin 3 (Sirt3) is a major mitochondrial deacetylase that is synthesized in the cytoplasm and imported into the mitochondrial matrix. It plays a key role in mitochondrial homeostasis by regulating mitochondrial protein acetylation levels and responding to cellular energy status [191]. It inhibits the ATX-LPA pathway to preserve β-cell identity and function. The depletion of Sirt3 enhances autotaxin (ATX) levels, leading to increased lysophosphatidic acid (LPA) production, which induces β-cell dedifferentiation and impairs insulin secretion. Inhibiting ATX in vivo mitigates β-cell dysfunction in HFD-fed mice lacking Sirt3, suggesting a potential therapeutic strategy for managing diabetes-associated β-cell dysfunction [192].

Sirt3 depletion is also attributed to the reduced deacetylation of the *FoxO1* gene and the subsequent decrease in its transcriptional activity. This suggests that Sirt3 downregulation contributes to β-cell dedifferentiation via *FoxO1* [120].

CBP/p300, acting as transcriptional co-activators and histone acetyltransferases, are crucial for β-cell function. Treatment with A-485, a selective inhibitor of CBP/p300 HAT activity, impairs insulin secretion and downregulates β- and α-cell identity genes without inducing dedifferentiation. A-485 also affects genes related to glucose sensing but not glycolysis. In prediabetic db/db mice, decreased CBP/p300 levels impact β-cell function genes, possibly involving histone H3K27 deacetylation and transcription factors like *Hnf1α* and *FoxO1*. This highlights the critical role of CBP/p300 HAT in maintaining β-cell identity [193].

### 6.3. Histone Methylation

Histone H3 lysine 4 (H3K4) methylation is crucial for sustaining gene expression related to insulin production and glucose responsiveness, with deficient methylation leading to an altered epigenome profile. Moreover, in diabetic models, there is a shift favoring inactive genes, potentially contributing to β-cell dysfunction [194].

Utilizing GSK126 and Tazemetostat (Taz) inhibitors of histone methyltransferase EZH2, researchers induced a phenotypic shift towards a β-like cell identity in exocrine cells from T1D donors. This transition was facilitated by chromatin modifications, particularly H3K27me3 and H3K4me3, suggesting EZH2 to be pivotal regulator of β-cells’ regenerative potential. Reprogrammed pancreatic ductal cells demonstrated insulin production and secretion in response to physiological glucose levels ex vivo [195].

### 6.4. DNA Methylation

Proper DNA methylation patterns are required to maintain the mature β-cell identity. Disruption of these methylation dynamics contributes to β-cell dedifferentiation in diabetes [196].

DNA methylation plays a significant role in regulating tyrosine hydroxylase (TH), an enzyme known to suppress insulin secretion, during β-cell differentiation. The ablation of the de novo DNA methyltransferase *Dnmt3a* in embryonic progenitors increases TH+ β-cells, reducing insulin secretion. However, postnatal β-cell-specific *Dnmt3a* ablation does not have the same effect. This suggests that maintaining a lower proportion of TH+ β-cells is vital for proper insulin secretion and that embryonic DNA methylation patterns regulate β-cell heterogeneity. Chronic overnutrition can lead to a loss of TH promoter methylation, increasing TH+ β-cells and compromising β-cell function, linking embryonic epigenetic regulation to postnatal β-cell identity and diabetes risk [197].

### 6.5. Chromatin Remodeling

Chromatin remodeling events play a crucial role in facilitating β-cell plasticity by regulating the expression of lineage-specific transcription factors and epigenetic modifiers.

The ARID1A subunit of the SWI/SNF chromatin remodeling complex regulates β-cell regeneration, with its expression naturally decreasing during β-cell proliferation. *ARID1A* knockout mice show increased protection against diabetes, indicating its role in regeneration. The specific deletion of Arid1a in β-cells enhances regeneration via neuregulin–ERBB–NR4A signaling. Blocking ERBB or NR4A1 disrupts this regeneration, suggesting that mSWI/SNF complex activity limits β-cell regeneration in health and disease [198].

It has also been found that Pdx1 recruits Swi/Snf to regulate pancreas development and β-cell function by influencing gene expression and chromatin structure. The loss of Swi/Snf in β-cells leads to reduced insulin production and impaired β-cell maturation, highlighting its crucial role. The interaction between Pdx1 and Swi/Snf is vital for β-cell proliferation and the expression of insulin-related genes [199].

Chromodomain Helicase DNA-Binding Protein 4 (Chd4), a subunit of the nucleosome remodeling and deacetylase complex, is found to be crucial for maintaining β-cell identity and function. An inducible β-cell-specific *Chd4* knockout mouse model showed that the loss of Chd4 led to glucose intolerance and impaired insulin secretion. Chd4-deficient β-cells exhibited an increased ratio of immature-to-mature insulin granules and elevated proinsulin levels. RNA sequencing revealed that *Chd4* deficiency altered chromatin accessibility and the expressions of key β-cell genes like *MafA*, *Glut2*, *Chga*, and *Chgb*. Similar defects were observed in human β-cells with *Chd44* knockdown, underscoring its importance in β-cell function. Additionally, it has been found that Chd4 is involved in the transcriptional activity of Pdx1 [200,201].

### 6.6. MicroRNAs in Pancreatic β-Cell Function and Identity

MicroRNAs (miRNAs) are critical for regulating many cellular functions, such as insulin signaling and glucose metabolism. Pancreatic β-cells depend on a network of miRNAs and their target mRNAs, which influence insulin production, secretion, cell growth, differentiation, survival, and apoptosis. Important miRNAs, including miR-7, miR-9, miR-375, miR-130, and miR-124, are abundant in β-cells and significantly impact their function. In diabetes, shifts in miRNA expression can lead to various complications such as β-cell dedifferentiation and transdifferentiation [202,203]. Table 2 provides detailed information on the impacts of various key miRNAs on β-cell dedifferentiation, transdifferentiation, neogenesis, and proliferation.

During the birth-to-weaning period, pancreatic β-cell growth and maturation are influenced by epigenetic programs activated by nutrients in human milk, including milk-derived exosomes (MEXs). These MEXs, containing miRNAs, drive β-cell proliferation, while reducing differentiation and mitochondrial function. Changes in MEX miRNAs during weaning may trigger β-cell differentiation, yet continuous exposure to bovine MEX miRNAs via cow’s milk could lead to β-cell dedifferentiation in adults. This suggests a potential link between prolonged bovine MEX exposure and an increased risk of diabetes [204].

Pancreatic β-cells undergo distinct metabolic changes during breastfeeding. Initially, they exhibit an immature state with high proliferation driven by mTORC1 activation and AMPK suppression. Upon weaning, β-cells transition to a mature state with reduced proliferation and altered mTORC1/AMPK activity, facilitating glucose-stimulated insulin secretion.

**Table 2:** miRNAs involved in β-cell dedifferentiation, transdifferentiation, neogenesis, or proliferation (De/Tra/Pro/Neo).

miRNA	Affected Genes	De/Tra/Pro/Neo	Type of Impact	References
*miRNA-148a*	*Pten*, *Ampk*	De	P	[205]
*microRNA-483*	*Aldh1a3*, *Socs3*	De	N	[206,207,208]
*miR-29-a*	*Cdc42*, *Irs1*, *AKT*	De	P	[209,210]
		Pro	N	
*miR-23a*	*SDF-1α*	Tra	N	[211]
*miR-24*	*Ire1α*, *MafA*	De	P	[212]
*miR-7*	*Pdx1*, *Isl1*	Neo	P	[213,214]
*miR-25/miR-92b*	*Neurod1*, *Mcl1*	De	P	[215]
*miR-9*	*Stxbp1*	De/Tra	P	[216]
*miR-96*	*PAK1*	β-cell dysfunction	N	[217]
*miR-33a-5p*	*Abca1*	De	Positive and negative	[218]
*miR-302s*	*NeuroD1 and Kat2b*	De	P	[203]
*miR-203*	*Zbtb20*	De	P	[219]
*miR-802*	*NeuroD1*, *Fzd5*	De	P	[220]
*miR-30d*	*Socs3*, *Glp1r*, *Igf1r*, *MafA*, *Pdx1*, *Nkx6.1*	Tra	P	[221]
*miR-212/132*	*Fbw7*	Neo	P	[222]
*miR-690*	*Sox9*, *Ngn3*, *Pdx1*	Neo	N	[223]
*miR-223*	*FoxO1*, *Sox6*	Pro	P	[121]
*miR-155*	*Pdx1*	De	P	[224]
*miR-153*	*SNAREs*, *MafA*, *NeuroD1*, *Bcl2*, *Ero1lb*, *Igf1r*, *Irs2*	De	P	[225]
*miR-320a*	*MafF*	De	P	[226]
*miR-17-92 cluster*	*Pten*, *Cdkn1a*, *p57*, *Bcl2L11*	Pro	P	[227]
*miR-21*	*Tgfb2*, *Fgfr3*, *Smad2*	De	P	[228,229]
*miR-184-3p*	*Crtc1*, *Slc25A2*	De and loss	P	[230]
*miR-199b-5p*	*Mlk3*	Pro	P	[231]
*miR-212-5p*	*Sirt2*	Insulin secretion and function	N	[232]
*miR-375*	*Motn*, *Mapkap 1*, *Pdx1*, *Pdpk1*, *Notch2*, *Hnf1β*, *Cadm1*, *Pax6*, *Crem*	De	N	[233,234,235,236,237,238,239]
		Pro	P	
		Neo	P	
*miR-200*	*Zeb1*	De	P	[240]
*miR-146a*	*Numb*	De	N	[241]
*miR-195*	*Mfn2*	De	P	[242]

The table is divided into five columns: “miRNA” lists the names of the miRNAs involved in β-cell De/Tra/Neo/Pro. “Affected Genes” describes the genes regulated by these miRNAs. “De/Tra/Neo/Pro” indicates the specific processes of β-cell plasticity and regeneration influenced by the miRNAs. “Type of Impact” describes whether the miRNAs have a positive or negative effect on β-cell plasticity and regeneration. “References” lists the sources used for each miRNA’s explanation. Abbreviations: P: Positive, N: Negative.

### 6.7. Long Non-Coding RNAs (LncRNAs)

Long non-coding RNAs (LncRNAs) have become crucial regulators of gene expression and cellular activity in pancreatic β-cells, profoundly influencing their function, adaptability, and growth. Recent studies have revealed several LncRNAs that are vital for preserving β-cell identity, controlling β-cell mass, and facilitating β-cell proliferation and regeneration. These discoveries highlight the significance of LncRNAs in β-cell biology and their potential as targets for diabetes treatment. Table 3 provides a summary of these key LncRNAs and their roles in β-cell regulation.

The table is organized into five columns: “LncRNAs” lists the names of the lncRNAs involved in β-cell De/Tra/Neo/Pro. “Direct and Indirect Targets” describes the miRNAs and genes regulated by the lncRNAs. “De/Tra/Neo/Pro” specifies the particular processes of β-cell plasticity and regeneration affected by the LncRNAs. “Type of effect” indicates whether the LncRNAs have a positive or negative effect on β-cell plasticity and regeneration. “References” provides the sources used for the information. Abbreviations: P: Positive, N: Negative.

## 7. Reprogramming and Neogenesis of β-Cells

Evidence suggests that newly emerged cells following β-cell destruction differ from original β-cells, demonstrating bihormonal activity by simultaneously producing both somatostatin and insulin. This dual hormone production may contribute to the management of hyperglycemia. Their rapid appearance following β-cell destruction, regardless of age, indicates a promising avenue for further investigation. Moreover, research indicates that these cells likely originate from Somatostatin 1.1 (sst1.1) δ-cells with a β-cell pre-identity, offering a potential source for β-cell regeneration after β-cell destruction [262]. Using single-cell transcriptomics in zebrafish during diabetes induction and recovery, researchers identified two distinct somatostatin-producing δ-cells, denoted as δ1 and δ2. Additionally, a small subset of glucose-responsive islet cells displayed characteristics of both β- and δ1-cells. These hybrid cells emerged spontaneously and gained glucose responsiveness, crucial for insulin expression during recovery. Furthermore, the overexpression of *Dickkopf-related protein 3* (*DKK3*), a member of the Dickkopf (DKK) family involved in Wnt signaling pathway regulation, enhanced the formation of these hybrid cells without harming β-cells [263].

The abundance of these δ-cells, as observed in T1D, could potentially result from α- to β- to δ-cell conversion [264,265]. Supporting this notion, it has been discovered that α-cells could also contribute to β-cell regeneration [264,266,267]. α-cells, especially those secreting glucagon, have the capacity to spontaneously transform into insulin-producing cells, thereby aiding in diabetes recovery, particularly post-puberty. A novel mechanism, partially governed by FoxO1, facilitates this transformation of somatostatin-producing δ-cells, particularly in juvenile individuals [266]. Additionally, a novel β-cell progenitor type displaying mesenchymal characteristics and expressing α-cell markers has been identified, potentially influencing β-cell regeneration in STZ-treated mice. These new β-like cells emerged within 48 h and, initially, were unresponsive to glucose. Insulin treatment normalized the glucose levels, preserving the neogenic β-like cells and gradually maturing the islet function. Intermediate cells expressing vimentin and *MafB* appeared within 16 h, serving as a major source of insulin-producing cells at 24 h [268].

In mice, α-cells and δ-cells can transform into insulin-producing β-cells after β-cell ablation, aiding in diabetes recovery, but whether this occurs in humans is unclear. This study demonstrates that non-β-cells from deceased human donors, including α-cells and γ-cells, can be reprogrammed to produce insulin using the transcription factors Pdx1 and MafA. Transplanted human α-cells reverse diabetes in mice and sustain insulin production for at least six months, retaining their original cell identity markers [269].

In addition to δ- and α-cells, scientists also discovered that ghrelin-producing ε-cells contribute to β-cell regeneration following significant β-cell loss. Augmenting ghrelin levels or expanding ε-cell populations supports this regeneration [270]. 

Beyond the conventional pancreatic cell types like α-, δ-, and ε-cells, ductal cells also play a role in contributing to the β-cell population, especially under diabetic conditions. 

Investigations revealed that there are two distinct niches for β-cell neogenesis: “β duct cells” near ducts and “β vessel cells” near blood vessels. Single-cell transcriptomic analysis revealed five unique populations among newborn β-cells, shedding light on potential regulatory mechanisms, including the role of *MafB*-expressing cells in β-cell specification. Additionally, comparative analysis with human embryonic stem cell-derived β-like cells highlighted similarities in the transcriptional profiles, suggesting shared regulatory pathways in β-cell maturation between mice and humans [271]. 

It is important to note that the evidence suggests that β-cell transdifferentiation plays a larger role in regenerating β-cells following extreme conditions like partial pancreatectomy, pancreatic duct ligation, and β-cell ablation with STZ, mimicking T1D, compared to neogenesis [264]. Conversely, β-cell neogenesis is more prominent in regenerating β-cells during insulin resistance in T2D, where an increase in β-cell mass is needed to counter hyperglycemia [272].

The process of neogenesis from β duct cells involves the expression of the endocrine master transcription factor Ngn3. Lineage tracing and single-cell sequencing (scRNA-seq) have unveiled that duct-derived somatostatin-expressing cells undergo a transition into β-cells, signifying a physiological mechanism for β-cell maintenance beyond mere proliferation. Moreover, the study has noted the presence of bihormonal cells within ducts, suggesting a ductal origin for certain islet cells and the potential for differentiation into various endocrine cell types. This underscores the significance of ductal cells in pancreatic regeneration and β-cell neogenesis [273].

The inhibition of MAP kinase-interacting serine/threonine kinase 2 (MNK2) with the small molecule CID661578 has been identified as a potent stimulator of β-cell regeneration. This small molecule facilitated β-cell neogenesis from ductal cells across various models, including zebrafish and human pancreatic organoids, by enhancing protein synthesis via modulation of the eIF4E-eIF4G interaction. MNK2 inhibition by CID661578 augmented eIF4E phosphorylation, promoting hypertranslation and β-cell regeneration, highlighting a potential therapeutic avenue for diabetes management [274].

## 8. Mitigation of β-Cell Dedifferentiation and Transdifferentiation

β-cell dedifferentiation and transdifferentiation can be reversed through various treatments, including dietary supplements, natural products, dietary interventions, hormones, and antidiabetic medications. These approaches offer promising strategies to restore β-cell function and mitigate diabetes.

### 8.1. Dietary Supplements

Glutathione is a crucial antioxidant synthesized within cells, playing a vital role in maintaining cellular health and defending against oxidative stress. Prolonged exposure of rats to long-term oscillating glucose (LOsG) triggers β-cell dysfunction through ROS stress. This stress disrupts FoxO1 signaling, leading to an elevation in thioredoxin interacting protein (TXNIP) levels and a reduction in insulin and *SOD-2* expression. Consequently, β-cell dedifferentiation and dysfunction occur. However, the timely administration of glutathione effectively prevents LOsG-induced β-cell failure [275].

Vitamin D3 therapy may offer a promising avenue for attenuating β-cell dedifferentiation. Research indicates that the administration of vitamin D3 and its analogues to the high-glucose-induced mouse insulinoma cell line (MIN6) results in the upregulation of the vitamin D3 receptor. This upregulation enhances β-cell function and maintenance by increasing insulin release and upregulating key transcription factors such as *Pdx1* and *MafA*. Consequently, there is an observed augmentation in the expressions of *Ins-1* and *Ins-2* genes [276]. 

Omega-3 polyunsaturated fatty acids (PUFAs) are known to have anti-hyperglycemic effects. Transgenic mice (mfat-1) that overexpress an enzyme converting omega-6 to omega-3 polyunsaturated fatty acids (*PUFAs*) were found to have significantly higher levels of eicosapentaenoic acid (EPA) in pancreatic tissues compared to wild-type mice. After STZ treatment, these transgenic mice did not develop diabetes and maintained normal insulin and blood glucose levels. The EPA metabolites from the CYP 450 pathway promoted insulin expression and increased β-cell markers in α-cells. This suggests that omega-3 PUFAs and their metabolites can promote α-to-β-cell transdifferentiation [277].

Folinic acid treatment, or the overexpression of *folate receptor 1* (*folr1*), has been shown to promote β-cell differentiation in a zebrafish model of β-cell loss. This effect extends to cultures of neonatal pig islets, suggesting its potential applicability in mammals. Notably, the source of increased β-cell differentiation in both zebrafish and pig islets was traced back to ductal cells. Additionally, comparative metabolomic analysis suggested that the regenerative effect of folinic acid might be linked to alterations in the pyrimidine, carnitine, and serine pathways [278].

Treatment with nicotinamide (NA), also known as niacinamide, a form of vitamin B3, significantly increased the number of insulin/PCNA, insulin/glucagon, and insulin/somatostatin double-positive cells compared to untreated groups. Notably, NA-treated rats showed enhanced insulin and *Pdx1*-positive cells, located within small clusters or scattered throughout the exocrine tissue and around ducts. Additionally, NA treatment increased *Notch1* expression while decreasing caspase-3 and TUNEL-positive β-cell numbers, suggesting that NA induces β-cell regeneration through ductal progenitor cell differentiation, acinar cell transdifferentiation, and the activation of Notch signaling [279].

Cathelicidin-based peptides like LLKKK18, a class of peptides derived from the cathelicidin family of antimicrobial peptides, have shown promise for T1D therapy. When delivered via poly (lactic-co-glycolic acid) (PLGA) nanoparticles (NPs), these peptides enhance β-cell function and neogenesis. In vitro studies have demonstrated glucose-stimulated insulin release and strong NP association with β-cells. In diabetic rats, these NPs effectively reduced hyperglycemia, improved pancreatic insulin content and glucose tolerance, and notably increased β-cell mass [280].

### 8.2. Dietary Interventions and β-Cell Function, Dedifferentiation, and Transdifferentiation

Intermittent fasting and calorie restriction have been found to regulate not only blood glucose and insulin levels, triglyceride and cholesterol levels, and insulin resistance but also insulin secretion, GSIS, and the modulation of β-cell transcription factors such as Pdx1, Nkx6.1, and MafA in obesity-induced diabetes [281]. 

Intermittent protein restriction (IPR) has also been found to significantly reduce hyperglycemia, protect pancreatic islets, and increase β-cell proliferation in diabetic Zucker fatty rats. IPR has been shown to reverse β-cell dedifferentiation, as evidenced by decreased expressions of dedifferentiation markers like *Cd81* and stress-related genes, while increasing markers of β-cell function such as islet amyloid polypeptide and chromogranin. Furthermore, IPR altered the expression of transcription factors, mitigating diabetes-associated changes in D-box binding PAR bZIP transcription factor (Dbp) and immediate-early response genes [282].

The ketogenic diet has also been shown to improve fasting blood glucose and insulin levels, β-cell mass, and identity by restoring the β/α-cell ratio, rearranging β-cells in the pancreas, and modulating β-cell transcription factors in diabetic mice [283].

A Chinese medical nutrition therapy incorporates medicinal herbs, whole grains, and intermittent energy restriction. It has demonstrated effectiveness in alleviating T2D. This therapy reduces fasting blood glucose levels, improves insulin sensitivity, enhances insulin production and secretion, modulates the gut microbiota, reduces pancreatic macrophage infiltration, and restores β-cell identity and morphology [284]. 

Increasing dietary fiber intake has been demonstrated to alleviate the progression of T2D and β-cell pathophysiological alterations, underscoring the therapeutic potential of dietary interventions. This is supported by research indicating that dietary fiber intake can safeguard and enhance insulin secretion through its influence on the intestinal microbiome. Specifically, it encourages the proliferation of short-chain fatty acid (SCFA)-producing strains and enhances the production of GLP-1, a hormone that stimulates insulin secretion [285].

### 8.3. Physical Activity and β-Cell Plasticity

Physical activity has been demonstrated to enhance pancreatic β-cell function and may play a crucial role in preventing or reversing β-cell dedifferentiation in the context of T2D. Exercise promotes β-cell function in T2D patients by increasing β-cell mass, improving glucose sensing, enhancing insulin secretion, and augmenting insulin content [285,286]. The protective effects of exercise on β-cells are mediated through multiple mechanisms: 1. Reducing metabolic stress: Physical activity facilitates increased glucose uptake in peripheral tissues, thereby lowering blood glucose levels and reducing the metabolic load on β-cells [287]; 2. Alleviating ER and oxidative stress: Exercise may attenuate ER stress and oxidative stress in β-cells, which are critical contributors to β-cell dysfunction; and 3. Modulating inflammation: Physical activity has the potential to modulate the immune environment of the pancreas, thereby reducing inflammatory processes that contribute to β-cell damage [286]. IL-6, which is produced by muscle cells during contraction, is associated with elevated levels of IL-1RA, IL-10, and GLP-1, which regulate abdominal fat lipolysis and promote β-cell protection, growth, and mass [288,289]. Additionally, certain myokines like angiopoietin and osteoprotegerin, specifically produced by triceps, exhibit anti-inflammatory effects [290]. Exercise can also increase levels of adiponectin, an adipokine synthesized by adipose tissue with anti-inflammatory properties [291]. Adiponectin has been shown to induce a shift of M1 (proinflammatory) macrophages to the M2 (anti-inflammatory) phenotype by reducing proinflammatory cytokines, including TNFα [292]. Notably, inflammatory responses by macrophages resident in the pancreas play a critical role in the fate of β-cells [3]. 

Besides IL-6, exercise also positively influences brain-derived neurotrophic factor (BDNF) [293] and C-X3-C motif chemokine ligand 1 (CX3CL1) [294]. Similar to GLP-1, these factors have beneficial effects on insulin secretion, glucose-stimulated insulin secretion (GSIS), and β-cell function [293,294].

Four exerkines elevated during exercise known to enhance β-cell proliferation, function, and maintenance include the following: irisin, an exerkine released by muscle tissue; follistatin, which is induced by exercise and secreted by the liver and muscles; osteocalcin, which is produced by bone marrow; and FGF21, which is produced snd decreted by hepatic tissues [295,296,297,298,299,300].

β-cell dedifferentiation and exercise: Although direct evidence linking exercise to the prevention or reversal of β-cell dedifferentiation is limited, various studies suggest that exercise may support the maintenance of β-cell identity and function. In animal models, voluntary running has been shown to prevent β-cell failure in susceptible islets [301]. Apelin, an exerkine that is involved in various physiological processes and is produced and secreted by various tissues, including adipose tissue, the heart, and skeletal muscle, has been shown to reverse β-cell dedifferentiation [302]. Exercise is also linked to the activation of the GABAergic system [303]. There is evidence suggesting that GABA and the activation of the GABAergic system might trigger the conversion of α-cells into β-cells, presenting a potential treatment avenue for Type 1 Diabetes (T1D) [304,305]. 

Combined effects of diet and exercise: The synergistic effects of dietary intervention and physical exercise on β-cell health are notable. Both strategies can alleviate metabolic stress on β-cells, and their combination, particularly through diet-induced weight loss, may enhance the benefits of each intervention. This dual approach has the potential to mitigate the adverse effects of T2D and β-cell identity [286].

Muscle mass reduction can significantly impact glucose metabolism and increase the risk of diabetes. Since approximately 80% of glucose clearance occurs in muscle tissue under normal glycemic conditions, any decrease in muscle mass or quality can adversely affect blood glucose regulation, leading to hyperglycemia, insulin resistance, metabolic stress, and impaired β-cell function and maintenance. Consequently, a correlation has been observed between sarcopenia (age-related loss of muscle mass and function) and the incidence of T2D. To address this issue, dietary supplements containing specific amino acids such as HMB (β-hydroxy β-methylbutyrate), leucine, glutamine, and arginine have been proposed as potential interventions. These supplements may help prevent sarcopenia and, consequently, reduce the risk of developing insulin resistance and T2D. By maintaining muscle mass and function, these amino acids could support better glucose regulation and metabolic health, particularly in aging populations or those at risk for muscle loss [306].

### 8.4. Antidiabetic Medications

#### 8.4.1. Glucagon-like Peptide-1 Receptor Agonist (GLP-1 RA)

Liraglutide, classified as a GLP-1 RA, stimulates GSIS. Sitagliptin, categorized as a dipeptidyl peptidase-4 inhibitor (DPP-4 inhibitor), elevates endogenous GLP-1 and glucose-dependent insulinotropic peptide (GIP) levels. These actions enhance insulin secretion and inhibit glucagon release. Both liraglutide and sitagliptin treatments have demonstrated the ability to reverse β-cell dedifferentiation in STZ-, high-fat-feeding- or HC-induced mice. Moreover, liraglutide administration has been observed to attenuate β-cell transdifferentiation into α-cells [307].

#### 8.4.2. Sodium–Glucose Co-Transporter 2 (SGLT2) Inhibitors

Dapagliflozin, a medication employed in the treatment of T2D, falls within the category of sodium–glucose co-transporter 2 (SGLT2) inhibitors. Emerging evidence suggests that dapagliflozin administration might mitigate transdifferentiation in diabetic mouse models. Interestingly, this effect appears to occur through a mechanism independent of reducing ambient blood glucose levels [308].

Luseogliflozin, another SGLT2 inhibitor, was tested on the pancreatic β-cell mass in db/db mice of various ages. The 6-week-old mice treated with luseogliflozin showed a significant increase in β-cell mass and upregulation of key genes compared to controls. While β-cell mass declined with age in the control group, luseogliflozin preserved the β-cell mass, especially in the early stages of diabetes. Immunohistochemistry and gene expression analyses supported these findings [309].

#### 8.4.3. Dipeptidyl Peptidase-4 Inhibitor (DPP-4 Inhibitor)

DPP-4 inhibitor Sitagliptin, known for its β-cell regenerative and immunoregulatory properties, and melatonin, a hormone primarily produced by the pineal gland that regulates sleep–wake cycles and possesses antioxidant, anti-inflammatory, and potential immunomodulatory properties, were studied for their combined effect on β-cell regeneration under glucotoxic stress in diabetic mouse models and humanized islet transplant models. The combination therapy demonstrated additive effects in promoting β-cell regeneration, reducing apoptosis, and improving glucose tolerance. In young diabetic mice, monotherapies induced β-cell transdifferentiation, while in older mice, they promoted β-cell proliferation and reduced apoptosis. Overall, the combination therapy proved superior, suggesting significant potential for future diabetes treatment [310].

### 8.5. Hormones

Hormones such as insulin, glucagon, and somatostatin play a crucial role in regulating β-cell dedifferentiation and transdifferentiation processes [190,311]. 

Glucagon has been identified as a key factor triggering the dedifferentiation of β-cells, with FoxO1 playing a partial role in this process. This interaction sheds light on the intriguing interplay between α- and β-cells. Blocking the glucagon receptor with a monoclonal antibody (mAb) has been found to reverse the effects induced by glucagon. Consequently, this intervention leads to improvements in β-cell size and function, while also preventing dedifferentiation in mice with T2D. Conversely, the inhibition of FoxO1 mimics the effects of glucagon, whereas its overexpression counteracts dedifferentiation [123]

Gastrin, a hormone primarily secreted by G cells in the stomach and duodenum, plays a key role in stimulating gastric acid secretion to aid in digestion. Recent studies have revealed that gastrin administration can induce the transformation and reprogramming of regenerative ductal cells in rats with 95% pancreatectomy, leading to the enhanced formation and expansion of β-cells. This process appears to involve the downregulation of *keratin 20* and *β-catenin*, representative of ductal dedifferentiation, and the upregulation of *Ngn 3* and *Nkx6-1*, indicating an endocrine progenitor phenotype [312].

The long-acting xenin analogue xenin-25[Lys13 PAL], a modified form of xenin-25 and peptide hormone that influences gastrointestinal function and glucose metabolism, has antidiabetic effects, promoting insulin secretion and improving pancreatic islet architecture. In high-fat-fed and STZ-induced insulin-deficient mice, xenin-25[Lys13 PAL] treatment reversed STZ-induced body weight loss, increased insulin levels, and reduced blood glucose. It also decreased pancreatic α-cell areas, reduced β-cell apoptosis, and promoted β-cell growth. Additionally, xenin-25[Lys13 PAL] decreased the transition of insulin-positive to glucagon-positive cells, suggesting that it helps maintain β-cell identity and function [313].

Administering T3, a thyroid hormone variant, efficiently counteracts the harmful effects of β-cell dedifferentiation and transdifferentiation. This corrective action not only boosts insulin secretion and restores vital markers but also safeguards pancreatic islet structure by halting β-cell polyploidization. T3 treatment has been shown to diminish the transformation of β-cells into α-cells, thus upholding insulin levels. Conversely, blocking T3 exacerbates transdifferentiation [314].

Angiotensin (1–7) (Ang (1–7)), a peptide hormone derived from the cleavage of Angiotensin II by enzymes such as ACE2, has demonstrated potential in mitigating high-glucose-induced apoptosis and dedifferentiation of pancreatic β-cells through its interaction with MAS receptors. In the presence of high glucose levels, the β-cell morphology deteriorates, insulin secretion diminishes, and markers associated with mature β-cells (*Pdx1* and *MafA*) decrease, while those linked to endocrine progenitor cells (*Oct4* and *Nanog*) increase. However, treatment with Ang (1–7) significantly reverses these detrimental changes. This reversal is attributed to the specific interaction between Ang (1–7) and its MAS receptors, which subsequently impacts the Wnt/β-catenin/FoxO1 pathway, ultimately safeguarding the β-cell function and identity against the adverse effects of high glucose [315].

Adipsin, also referred to as complement factor D, is a serine protease enzyme encoded by the ADIPOQ gene. Its pivotal role lies within the alternative complement pathway, an integral part of the innate immune system. Predominantly synthesized by adipocytes, adipsin actively participates in adipose tissue metabolism, exerting influence over energy regulation and adipocyte differentiation. Additionally, adipsin contributes to the generation of complement component C3a, which exhibits insulin secretagogue activity. Emerging evidence suggests that elevated levels of blood adipsin/C3a may uphold β-cell identity amidst diabetic conditions [316].

### 8.6. Natural Products

Sangzhi alkaloids (SZ-A), extracted from *Morus alba* L. (mulberry twig), have shown promise in mitigating hyperglycemia and glucose intolerance in diabetic mice. Moreover, they have been found to restore β-cell identity and function by modulating the levels of β-cell-specific transcription factors under diabetic conditions [317].

Eugenol (4-allyl-2-methoxyphenol) is a naturally derived compound present in clove, basil, cinnamon, and nutmeg. When administered to Ins-1 β-cells exposed to high glucose and lipid levels, eugenol demonstrated the ability to partially restore the expressions of certain β-cell-specific genes, including *Glut2*, *NeuroD1*, and *FoxO1* [318].

Piperine, a compound in black pepper, offers numerous health benefits, including anti-inflammatory, antioxidant, anti-obesity, and antidiabetic effects. In a mouse model of obesity, a 10-week piperine treatment improved glycolipid metabolism, insulin resistance, and metabolic inflammation. Piperine enhanced glucose metabolism, reduced inflammation, and prevented β-cell dedifferentiation while also decreasing M1-like macrophage polarization in islets and visceral adipose tissue [319].

Psilocybin, a natural compound from Psilocybe mushrooms, acts as an agonist for serotonin receptors. It has been shown to reduce β-cell loss induced by high-glucose–high-lipid (HG-HL) conditions by mitigating apoptosis, likely through modulating TXNIP and STAT pathways. Additionally, psilocybin affects key genes related to β-cell dedifferentiation, such as *Pou5f1* and *Nanog*, indicating its potential therapeutic role in protecting against β-cell dysfunction and dedifferentiation in T2D [320].

Phytocannabinoids are a group of secondary metabolites produced by *Cannabis sativa*. We recently studied the impacts of various phytocannabinoids on β-cell survival, function, and dedifferentiation in high-glucose–high-lipid-induced INS-1 β-cells. Our results indicate that certain phytocannabinoids can restore an impaired β-cell identity by reactivating β-cell-enriched transcription factors [321].

Gymnemic acid (GA) is a compound found in the leaves of *Gymnema sylvestre*, a plant native to India and Africa. It is known for its unique property of temporarily suppressing the taste of sweetness, making sweet foods taste less appealing. GA treatment demonstrated promising outcomes by maintaining normoglycemia in diabetic rats through enhanced insulin secretion via the upregulation of β-cell regeneration markers such as *Pdx1*, Neurognin 3, *MafA*, and *NeuroD1*. Additionally, GA facilitated proliferation by activating the PI3K/AKT pathway and promoting cell adhesion through the increased expression of ECAD and β-catenin, thereby preserving islet architecture [322].

Treatment with magainin-AM2, an analog of magainins, which are antimicrobial peptides originally discovered in the skin of the African clawed frog, *Xenopus laevis*, and growth hormone improved glucose tolerance and fasting blood glucose levels post-STZ treatment and increased the total cell counts and insulin+ and glucagon+ cells per islet. The expressions of key factors for α-to-β-like cell conversion and cell proliferation/differentiation were significantly upregulated, suggesting a potential for the pharmaceutical induction of α-cell production and their transdifferentiation to functional β-like cells, with magainin-AM2 showing the greatest efficacy [323].

The administration of a Fufang-Zhenzhu-Tiaozhi capsule (FTZ), a traditional Chinese medicine prescription commonly used in clinical practice, increased insulin levels, reduced glucose levels, and promoted β-cell regeneration in T1DM mice. Additionally, FTZ inhibited inflammatory cell invasion and islet cell apoptosis, preserving the quantity and quality of β-cells. The promotion of β-cell regeneration by FTZ was associated with the upregulation of key transcription factors (*Pdx1*, *MafA*, and *Ngn3*), suggesting FTZ as a potential therapeutic drug for T1DM [324].

### 8.7. The Role of the Gut Microbiome in β-Cell Plasticity

The gut microbiome appears to play an important role in shaping β-cell biology through bacterial metabolites, proteins, extracellular vesicles, and immunomodulatory effects [325].

Short-chain fatty acids (SCFAs) produced by gut bacteria help regulate metabolism. SCFAs such as sodium acetate, sodium propionate, and sodium butyrate (SB) did not immediately affect insulin release from pancreatic islets. However, prolonged exposure to SCFAs improved the function of β-cells. An analysis of RNA levels showed that SB-treated islets had lower expression levels of genes related to insulin secretion and β-cell identity, such as *Pdx1* and *MafA*. SB increased oxygen consumption but reduced glucose-stimulated oxygen consumption in islets, with no changes in genes related to glycolysis or the tricarboxylic acid cycle. Additionally, SB lowered the expression of *Kcnj11*, which encodes a channel involved in regulating insulin release, increased calcium levels, and promoted insulin gene expression by affecting the histone modification H3K18bu on its promoter. This suggests that while SB improves islet function, it also alters the expressions of genes related to β-cell identity [326].

Additionally, butyrate demonstrates anti-inflammatory properties, preserving the β-cell function against IL-1β-induced dysfunction. Butyrate exerts these effects through the suppression of IL-1β-induced inflammatory genes and nitric oxide production. Notably, butyrate inhibits NF-κB activity via histone deacetylase inhibition without affecting NF-κB nuclear translocation. These findings highlight butyrate’s potential in mitigating β-cell dysfunction through NF-κB pathway modulation, offering insights into T2D prevention and treatment strategies [327].

An altered gut microbiota is linked to T1D, influencing SCFAs and glucose homeostasis. A dietary supplement called HAMSAB, designed to increase serum acetate and butyrate, improved glycemic control in NOD mice and patients with established T1D. Single-cell RNA sequencing on islet cells from NOD mice fed a HAMSAB or control diet revealed that HAMSAB reduced immune-cell infiltration in the pancreas, preserved β-cell function and identity, and decreased cellular stress. This suggests that SCFAs have potential anti-inflammatory effects and that HAMSAB may be a promising approach to managing T1D [328].

### 8.8. Other Interventions

Following bariatric surgery, rats undergoing Roux-en-Y gastric bypass (RYGB) and sleeve gastrectomy exhibited significant improvements in metabolic parameters and an increase in pancreatic β-cell proportion compared to Sham-operated rats. Both the RYGB and Sleeve groups demonstrated a decrease in the α-to-β-cell ratio. The study identified Gcg+/Ppy+ and Ins+/Gcg+/Ppy+ multiple hormone-expressing cells post-surgery, suggesting potential transdifferentiation events. Notably, the discovery of Ins+/Gcg+/Ppy+ cells, displaying features of both α- and β-cells, implies a role for transdifferentiation in enhancing β-cell function following bariatric surgery [329].

## 9. Advancements in Stem Cell-Based Therapies for Diabetes Mellitus

Recent studies have detailed the generation of functional β-cells from both embryonic and induced pluripotent stem cells. Adipose-derived mesenchymal stem cells (Ad-MSCs) are potential candidates for generating insulin-producing cells (IPCs), irrespective of donor age, gender, or health status. These cells demonstrated insulin secretion in response to glucose and dysfunction with palmitate exposure. Gene expression analysis showed over 5000 differentially regulated genes, aligning closely with native β-cells. Pathway analysis confirmed relevance to stem cell differentiation and pancreatic development, indicating that adipose-derived β-cells may be a promising clinical therapy for diabetes [330].

Research indicates that manipulating the *FoxO1-1* gene during the final stages of differentiation may enhance IPC generation from Ad-MSCs. While FoxO1 is essential for early β-cell development, its overexpression in mature β-cells can cause glucose intolerance and hyperglycemia. Similarly, in Ad-MSCs, FoxO1 supports early differentiation, but reducing its expression at later stages results in more mature and functional IPCs [331].

In addition to Ad-MSCs, bone marrow-derived mesenchymal stem cells (bm-MSCs) can potentially be transdifferentiated into islet-like cells for treating T1DM. In a research study, this process was induced using high glucose, nicotinamide, β-mercaptoethanol, betacellulin, and IGF-1, and transdifferentiation was confirmed through glucose challenge assays and gene expression analysis. The transdifferentiated cells were then microencapsulated with 1% alginate and cultured in a fluidized-bed bioreactor to ensure stability and proliferation. After transplantation into the omentum of STZ-induced diabetic Wistar rats, blood glucose levels significantly decreased within a week, and the treated rats’ weight, insulin, and C-peptide levels returned to normal [332]. Moreover, there is evidence suggesting that exosomes derived from bm-MSCs, known as bm-MSC-derived exosomes (bmMDEs), could alleviate β-cell dedifferentiation and enhance their function in type II diabetic rats as well as primary islets exposed to high glucose levels. This therapeutic effect is facilitated by miR-146a, which acts on the NUMB/β-catenin signaling pathway [241].

A protocol for the transformation of human fibroblasts into endodermal cells using non-integrative episomal reprogramming factors, growth factors, and chemical compounds has been demonstrated. The differentiated cells undergo a progression from definitive endodermal progenitors to posterior foregut-like progenitors, then to pancreatic endodermal progenitors, with significant expansion potential. Through screening, specific compounds were identified that enhance the differentiation of pancreatic endodermal progenitors into functional β-like cells in laboratory settings. When transplanted, these β-like cells exhibit GSIS in animal models, offering protection against chemically induced diabetes [333].

Human umbilical cord mesenchymal stem cells (hUC-MSCs) have shown promising potential in reverting β-cell dedifferentiation when cocultured with purified human T2D mellitus (hT2DM) islets. This reversal is orchestrated through a targeted interaction facilitated by the secretion of interleukin-1 receptor antagonist (IL-1Ra) from hUC-MSCs. This mechanism plays a pivotal role in modulating the overall inflammatory responses within diabetic islets, highlighting the therapeutic significance of hUC-MSC-based interventions in diabetes management [334].

MSC secretomes, which are collections of molecules including proteins, cytokines, and other factors secreted by MSCs, show higher levels of total protein and IL-4 when derived from 3D cultures compared to 2D cultures. In vivo, these secretomes induce regulatory T cells, modulate cytokine release, and increase β-cell regeneration, particularly those from 3D cultures. This demonstrates the dual therapeutic potential of MSC secretomes in T1D, promoting both immunomodulation and β-cell regeneration. Culturing MSCs in 3D scaffolds enhances these effects, suggesting a promising approach for T1D therapy [335].

hTERT-MSC (human telomerase reverse transcriptase MSC) transplantation, both intrapancreatically (IPR) and intravenously (IVR), in pancreatectomized mice, leads to the increased proliferation of pancreatic β-cells. IPR demonstrates moderate advantages over IVR. These cells activate pancreatic progenitor cell characteristics through the AKT/Pdx1/FoxO1 signaling pathway and modulate growth factors and proinflammatory cytokines [119].

## 10. β-Cell Proliferation and Regeneration

β-cell regeneration and proliferation are essential for diabetes treatment, restoring insulin production, and managing blood glucose levels. Enhancing these processes can potentially reverse diabetes, reducing the need for external insulin therapy. Strategies include stimulating endogenous β-cell proliferation, neogenesis, and transdifferentiation using small molecules and transcription factors, as well as deriving β-cells from pluripotent stem cells [336,337].

### 10.1. The Suppression of Dual-Specificity Tyrosine-Regulated Kinase 1A (DYRK1A)

The DREAM complex plays a pivotal role in maintaining quiescence in human β-cells. Comprising transcriptionally repressive proteins, this complex forms in response to DYRK1A kinase activity, enforcing cellular quiescence. When DYRK1A activity is inhibited, DREAM subunits transition into the pro-proliferative MMB complex. Small molecule DYRK1A inhibitors stimulate replication in human β-cells by shifting the DREAM complex from its repressive state to the proliferative MMB configuration [338].

Small-scale suppression of DYRK1A holds promise for pharmaceutical intervention in β-cell regeneration for diabetes, addressing a significant therapeutic gap. However, due to DYRK1A’s involvement in critical signaling pathways, it is essential to maintain its levels similar to those found in healthy individuals. In silico tests have identified several plant-based compounds as potential ligands for β-cell regeneration. These compounds include 3-[6-(3-methyl-but-2-enyl)-1H-indolyl]-6-(3-methyl-but-2-enyl)-1H-indole, Lanceolatin B, Lysicamine, Pratorinine, Pratorimine, Lanceolatin A, Lanuginosine, Hippacine, (-)-Semiglabrin, Aegyptinone B, 3′-Prenylnaringenin, and 8-C-p-hydroxybenzylluteolin. Computational analyses suggest these compounds have good intestinal absorption and favorable drug-like properties, making them promising candidates for new medications, pending further in vivo validation [12].

Various synthetic small molecules have shown potential for promoting β-cell regeneration by inhibiting DYRK1A activity. AC27, a compound with superior selectivity compared to another DYRK1A inhibitor, harmine, not only inhibits DYRK1A but also reduces its cellular levels, resulting in increased β-cell proliferation and enhanced insulin and C-peptide secretion in response to glucose. These effects were stable over a 12-day period and persisted after the inhibitor was withdrawn. AC27’s benefits were validated in insulinoma cell lines, iPSC-derived β-cell organoids, and isolated mouse pancreatic islets, with its effects further enhanced by TGF-β inhibition [339]. 

Gua et al. conducted a chemical screen in zebrafish to identify small molecules that increase the number of incretin-expressing cells. One of the identified compounds, AZ Dyrk1B 33, was found to be a potent inhibitor of DYRKs and effectively boosted incretin+ cell populations in zebrafish. Moreover, it demonstrated improved glucose regulation in mouse models. The research suggests that AZ Dyrk1B 33 likely influences enteroendocrine cell differentiation through NFAT signaling pathways. Unlike the DYRK1A inhibitors known to enhance β-cell proliferation, AZ Dyrk1B 33 did not affect β-cell mass in this study, highlighting its specific role in enteroendocrine cells regulated by DYRK1B [340]. 

While DYRK1A inhibitors such as AC27 hold potential for enhancing β-cell proliferation and function by targeting β-cell cycle pathways, significant obstacles persist. These include achieving β-cell specificity, refining evaluation techniques, and overcoming the low proliferation rates of human β-cells. To address these issues, structural modifications are needed to improve targeting and minimize off-target effects. Furthermore, integrating DYRK1A inhibitors with other antidiabetic medications could enhance their therapeutic efficacy [340,341].

Common DYRK1A inhibitors can induce β-cell proliferation, but with low rates and limited specificity. It has been shown that combining any GLP1R agonist with any DYRK1A inhibitor results in a significant synergistic increase in human β-cell replication (5 to 6%), leading to an actual increase in human β-cell count. This combination did not cause β-cell dedifferentiation and was effective for both normal human β-cells and those derived from individuals with T2D [342].

Molecular docking analysis revealed diosmin, a flavonoid compound found in various plants such as *Zanthoxylum chalybeum* Engl., as a potential inhibitor of DYRK1A. In vitro experiments showed that the extract promoted cell viability and proliferation, particularly under conditions of palmitate exposure. However, in vivo results indicated only mild β-cell regenerative potential, suggesting further investigation is needed to understand the molecular interactions underlying these effects, particularly regarding diosmin’s role as a DYRK1A inhibitor [343].

### 10.2. Glucagon Receptor Antagonism in β-Cell Regeneration

Research indicates that the glucagon receptor antibody (anti-GCGR) has significant potential for promoting β-cell regeneration in diabetes models. Anti-GCGR treatment effectively reverses hyperglycemia, elevates plasma insulin levels, and restores β-cell mass through both β-cell proliferation and α-to-β-cell transdifferentiation [13,14,15,16]. Using lineage-tracing techniques, researchers found that GCGR mAb treatment causes α-cells to regress to progenitor states and reactivates Ngn3 progenitors, promoting their transdifferentiation into β-cells. This treatment also upregulates the genes associated with β-cell regeneration and improves insulin secretion in primary mouse islets [14].

Treatment using REMD 2.59, a competitive human GCGR antagonist, lowered blood glucose levels while elevating the plasma levels of glucagon and somatostatin. Notably, the administration of a GCGR monoclonal antibody substantially increased α-cell and δ-cell populations in both normal and T1D mice. This increase in δ-cell mass was linked to enhanced proliferation and neogenesis originating from pancreatic ducts. Remarkably, the observed δ-to-β-cell transdifferentiation in T1D mice post-treatment suggests a promising avenue for restoring β-cell numbers [344]. 

Additionally, it has been found that REMD 2.59 induces α-cell regression to progenitors, facilitating β-cell neogenesis and upregulating regeneration-related genes in mouse islets. This highlights the GCGR mAb’s potential for β-cell regeneration in T2D, supporting α-cell to β-cell conversion and progenitor-driven β-cell formation [345].

GCGR mAb treatment in PANIC-ATTAC mice, which triggers caspase-8–mediated apoptosis specifically within the pancreatic β-cell, stimulates α-cell to β-cell conversion, leading to sustained improvements in glycemic control and effectively curing diabetes in this model. While α-cell hyperplasia may pose challenges in T2D, in T1D, it offers a potential source for generating new insulin-producing cells [346]. 

FGF21 has been shown to play a crucial role in the regenerative effects of GCGR antagonism on β-cells. The levels of FGF21 increase in both the plasma and liver in response to GCGR antagonism. The inhibition of FGF21 activity diminishes the upregulation of β-cell identity genes induced by the GCGR mAb. Furthermore, the increases in the β-cell number and proliferation driven by the GCGR mAb are less pronounced in mice lacking FGF21 or hepatic FGF21 [16].

Interestingly, anti-GCGR-induced α-cell hyperplasia can occur independently of β-cell regeneration. Furthermore, this therapy has shown efficacy in non-human primates, and when combined with anti-CD3, it significantly enhances β-cell mass and reverses diabetes in T1D models [13].

GCGR antagonism also stimulates the secretion of glucagon and glucagon-like peptide 1 (GLP-1) [347,348], which promotes β-cell regeneration. Blocking GLP-1 receptor (GLP-1R) signaling reduces these regenerative effects, suggesting that GLP-1R activation by glucagon is crucial for the β-cell regenerative effects induced by the GCGR mAb [15].

GLP1 induces insulin–glucagon-positive cells in rat pancreatic islets, potentially indicating α- to β-cell transdifferentiation. Molecular analyses suggest that GLP1 promotes β-cell function through the PI3K/AKT/FOXO1 pathway, enhancing *Pdx1* and *MafA* expressions while reducing *MafB*, suggesting a mechanism for α-cell inhibition and β-cell promotion by GLP1 [349].

The effect of Liraglutide and the GCGR mAb in promoting pancreatic β-cell neogenesis in T1D mice has been examined. Liraglutide treatment expanded β-cell mass through self-replication, differentiation, and transdifferentiation from α-cells, while GCGR mAb treatment significantly increased β-cell mass. However, the combination of both drugs did not further enhance the β-cell area but reduced plasma glucagon levels and increased the proportion of β-cells to α-cells [350].

Dapagliflozin has been found to enhance the expression of pancreatic endocrine progenitor and β-cell markers, including *Pdx1*, in cultured primary rodent islets and αTC1.9 cells under hyperglycemic conditions. This regenerative effect is partially mediated by GLP-1 secreted from α-cells [351]. 

The stimulatory impact of dapagliflozin on GLP-1 secretion is attributed to the reshaping of the gut microbiota and the modulation of metabolites involved in tryptophan metabolism, particularly l-tryptophan, which augments GLP-1 secretion and insulin levels. These beneficial effects are mediated by gut microbiota and tryptophan metabolism and are attenuated by GLP-1 receptor antagonism or pancreatic *Glp1r* knockout [352].

The GLP-1–oestrogen conjugate yielded significant benefits in managing diabetes. The GLP-1–oestrogen conjugate notably reduced daily insulin needs by 60% and triggered the endoplasmic-reticulum-associated protein-degradation system. This intervention improved β-cell survival and regeneration. Moreover, the GLP-1–oestrogen conjugate shielded human β-cells from dysfunction induced by cytokines [353]. 

The impacts of γ-aminobutyric acid (GABA) alone or in combination with the GCGR mAb on β-cell and α-cell mass in T1D mice have also been studied. Treatment with GABA or the GCGR mAb individually improved hyperglycemia and increased β-cell mass, with similar effects observed in the combination treatment. Interestingly, the combined treatment attenuated GCGR mAb-induced α-cell hyperplasia [354]. 

In summary, anti-GCGR treatment and GCGR antagonism promote β-cell regeneration through multiple mechanisms, including α-to-β-cell transdifferentiation, the upregulation of β-cell genes, and involvement of GLP-1 and FGF21 pathways. These findings highlight the potential of GCGR antagonists in enhancing β-cell mass and function in diabetes therapy.

### 10.3. The Polyamine Biosynthesis Pathway

The polyamine biosynthesis pathway is a conserved metabolic process in all organisms, involving key enzymes like aminopropyltransferases (spermidine and spermine synthases). This pathway is essential for cellular functions, including growth, proliferation, and stress responses. Research has shown that inhibitors of polyamine biosynthesis, such as difluoromethylornithine (DFMO), enhance β-cell regeneration without affecting overall growth or pancreas length, while Imatinib mesylate, a tyrosine kinase inhibitor that inhibits polyamine biosynthesis pathway, has no impact. These findings suggest that targeting polyamine biosynthesis, especially with DFMO, holds promise for stimulating β-cell regeneration in diabetes [17].

In the context of the polyamine pathway, administering spermidine to NOD mice heightened the diabetes occurrence, despite unchanging insulitis levels. This elevation was correlated with increased pancreatic tissue polyamines and markers of autophagy. Remarkably, spermidine induced shifts in the T-cell composition within pancreatic lymph nodes, amplifying pro-inflammatory T-cell populations in diabetic mice and demonstrating differing impacts on regulatory T cells depending on disease onset, ultimately exacerbating T1D incidence [355].

In preclinical models, DFMO has also been shown to delay T1D onset by reducing β-cell stress. A clinical trial with recent-onset T1D patients showed that DFMO is safe and tolerable, reducing urinary putrescine levels and preserving C-peptide at higher doses. Transcriptomic and proteomic analyses suggest DFMO preserves β-cell function by altering mRNA translation, protein transport, and secretion in stressed islets [356].

### 10.4. Other Pathways Involved in β-Cell Proliferation

In addition to the aforementioned methods to induce β-cell proliferation and regeneration, there are other approaches, such as inhibiting TBK1/IKKε and salt-inducible kinases (SIKs).

Xu et al. found TBK1/IKKε inhibitors (TBK1/IKKε-Is) to be potent enhancers of β-cell regeneration in a zebrafish model of T1D, validated across mammalian systems including human and rat β-cells, and STZ-induced diabetic mice. The mechanism involves TBK1/IKKε inhibition, which promotes β-cell proliferation via the cAMP-PKA-mTOR signaling axis through PDE3, highlighting a novel role for TBK1/IKKε in β-cell mass modulation. Notably, TBK1/IKKε-Is like amlexanox and PIAA selectively enhance β-cell proliferation without inducing proliferation in other cell types, showing promise for targeted therapy [357].

An in vivo study by Charbord et al. identified HG-9-91-01(HG) as a β-cell mitogen that enhances β-cell proliferation in zebrafish, mouse, and human β-cells by inhibiting salt-inducible kinases (SIKs), specifically SIK1–SIK3, members of the AMPK-related protein kinase family. HG stimulates β-cell proliferation through UPR activation via ATF6–IRE1 pathways. Despite showing specificity for SIK1, HG’s pharmacokinetic limitations currently preclude its consideration as a drug candidate [358].

## 11. Conclusions

β-cell dedifferentiation and transdifferentiation are critical factors in the progression of both Type 1 and T2D (T1D and T2D). Metabolic stress and inflammation significantly contribute to β-cell dedifferentiation and transdifferentiation by dysregulating various pathways, including ER stress, the mTOR pathway, the endocannabinoid system, viral infections, and aging. Targeting these processes is considered a potential therapeutic strategy for diabetes. Various natural products, hormones, and dietary treatments have shown promise in mitigating β-cell dedifferentiation and transdifferentiation.

β-cell regeneration and neogenesis present potential treatments for diabetes, particularly T1D. Research has demonstrated that various pancreatic cells, including δ-, ε-, and α-cells, can participate in β-cell regeneration under diabetic conditions. Treatments such as glucagon receptor antagonism, both alone and in combination with GLP-1 agonism and DYRK1A inhibition, have been effective in regenerating ablated β-cells in diabetic models. Additionally, differentiating MSCs into β-cells, followed by the transplantation of these newly differentiated cells, offers a promising approach for diabetes treatment, especially for T1D. It has been shown that specific inducers containing certain components can effectively promote the neogenesis of MSCs into β-cells.

## 12. Limitations

1. Lack of review on experimental models: In this study, we did not review the experimental models and the methods used to model β-cell dedifferentiation and transdifferentiation. A comprehensive examination of the various approaches to model β-cell dedifferentiation and transdifferentiation in both in vitro and in vivo settings would be highly beneficial.
2. Limited scope on cell plasticity: This review primarily focused on β-cell plasticity and did not cover the plasticities of other cell types. It would be valuable to briefly explore the plasticities of other cells such as hepatocytes, hepatic stellate cells, epidermal stem cells, melanocytes, astrocytes, intestinal stem cells, cardiac fibroblasts, club cells, podocytes, and others. Investigating the plasticities of these diverse cell types could offer insights into the broader applications and implications of cellular plasticity.
3. Context of cell plasticity in other diseases: While this review centered on β-cell plasticity, it would have been beneficial to study cell plasticity in the context of other diseases such as liver diseases, neurodegenerative diseases, and cancer. Understanding how cellular plasticity manifests and contributes to the pathology of these diseases could inform the development of new therapeutic strategies and enhance our knowledge of disease progression and treatment.

## 13. Unanswered Questions

Even though the progress in diabetes research and the role of β-cell plasticity in it have been substantial, many questions have remained unanswered: 

Combinatorial effects of treatments: What are the combined impacts of various effective natural products and common antidiabetic medications on β-cell dedifferentiation, transdifferentiation, regeneration, and neogenesis?

Endocannabinoid system and β-cell plasticity: How does the endocannabinoid system influence β-cell plasticity? Can the modulation of this system induce β-cell regeneration and neogenesis?

Serotonin signaling pathway and β-cell plasticity: How does the serotonin signaling pathway affect β-cell plasticity? Can targeting this pathway enhance β-cell regeneration and neogenesis?

Long-term effects of β-cell plasticity interventions: What are the long-term effects of interventions aimed at promoting β-cell plasticity on the overall pancreatic function and glucose homeostasis?

## Figures and Tables

**Table 1:** Important transcription factors involved in β-cell dedifferentiation, transdifferentiation, neogenesis, or proliferation (De/Tra/Neo/Pro).

Transcription Factor	Full Name	Targets Genes	Impact on the Genes	De/Tra/Neo/Pro	Impact on β-Cell Plasticity/Regeneration	References
Pax4	Paired Box 4	*GCG*	R	De	N	[101,102,103,104]
		*Arx*	R			
		*Ins*	I	Tra	N	
		*Bcl-xL*	I			
		*Gherlin*	R	Neo	P	
		*SST*	R			
		*IAPP*	R	Pro	P	
		*C-myc*	I			
Nkx2.2	NK2 homeobox 2	*Ins*	I	De	N	[105,106,107,108,109,110]
		*Pax6*	I			
		*Nkx6-1*	I	Tra	N	
		*MafA*	I			
		*NeuroD1*	I	Neo	P	
		*Arx*	R			
		*SST*	R	Pro	NM	
		*Gherlin*	R			
Mnx1 (also known as HLXB9 or HB9)	Motor neuron and pancreas homeobox 1	*Nkx6.1*	I	De	N	[111,112,113,114,115]
				Tra	N	
		*Ins*	I	Neo	NM	
				Pro	P	
FoxO1	Forkhead box O1	*Ins1*	R/I	De	N and P	[116,117,118,119,120,121,122,123]
		*Pdx1*	R	Tra	N and P	
		*NeuroD*	I			
		*Superoxide dismutase 1 (Sod1)*	I	Neo	N	
		*Catalase (Cat)*	I			
		*MafA*	I	Pro	P	
		*Glutathione peroxidase 1*	I			
Pdx1	Pancreatic and Duodenal Homeobox 1	*Ins*	I	De	N	[124,125,126,127,128]
		*Glut2*	I			
		*Gck*	I	Tra	N	
		*IAPP*	I			
		*SST*	I	Neo	P	
		*Pdx1*	I			
		*Ngn3*	I	Pro	P	
		*NFκB*	R			
		*Maf* *A*	I			
MafA	Musculoaponeurotic Fibrosarcoma Oncogene Family, Protein A	*Ins*	I	De	N	[129,130,131,132,133,134,135]
		*Glut2*	I			
		*Gck*	I	Tra	N	
		*Cacng4*	I			
		*Nanog*	R	Neo	P	
		*Pdx1*	I			
		*NeuroD1*	I	Pro	P	
		*Nkx6.1*	I			
		*Glis3*	I			
Ngn3	Neurogenin 3	*NeuroD1*	I	De	N	[99,136,137,138,139]
		*Pax4*	I			
		*Pax6*	I	Tra	N	
		*Isl1*	I			
		*Cdkn1a*	I	Neo	P	
		*Nkx2-2*	I			
		*Insm1*	I	Pro	N	
		*Kcnj11*	I			
		*Gck*	I			
		*Slc30a8*	I			
		*Slc18a2*	I			
SOX9	Sex-determining region Y (SRY)-box 9	*Ngn* *3*	I	De	N	[140,141,142,143]
		*Nkx* *6.1*	I	Tra	N	
		*Pax6*	I	Neo	P	
		*Ins*	I	Pro	P	
HNF1A	Hepatocyte Nuclear Factor 1 A	*HNF4A*	I	De	N	[144,145,146,147,148,149]
		*AS1*	I			
		*Tmem27*	I			
		*Kcnj11*	I			
		*Glut2*	I	Tra	N	
		*Ins*	I	Ne	P	
		*NeuroDI*	I	Pro	P	
		*Gck*	I			
		*MafB*	I			
		*Gipr*	I			
		*Glpr1*	I			
		*G6pc2*	I			
		*Slc5a1*	I			
		*Pdx1*	I			
		*Bcl2li(Bcl-xL)*	I			
ARX	Aristaless-Related Homeobox	*Gcg*	I	De	p	[150,151,152,153]
		*Pax4*	R			
		*SST*	R	Tra	p	
				Neo	N	
				Pro		
GATA6	GATA Binding Protein 6	*iNOS*	R	De	N	[154,155,156,157,158]
		*Ins*	I			
		*Glut2*	I	Tra	N	
		*Abcc8*	I			
		*Snap-25*	I	Neo	P	
		*Pdx1*	I			
		*Nkx6.1*	I	Pro	P	
		*MafA*	I			
GLIS3	GLI Similar 3	*Ins*	I	De	N	[159,160,161,162,163]
		*Ngn3*	I	Tra	N	
		*MafA*	I	Neo	P	
		*Glut2*	I	Pro	P	
PAX6	Paired Box 6	*Ins*	I	De	N/P	[164,165,166,167]
		*GCG*	R			
		*Glut2*	I			
		*Gipr*	I			
		*Nkx2.2*	I			
		*PC1/3*	I			
		*Glp1r*	I			
		*SST*	R	Tra	N/P	
		*Gherlin*	R			
		*Isi*	R	Neo	P	
		*Foxa2*	R/I			
		*Ngn3*	R/I	Pro	P	
		*Pdx1*	R/I			
		*NeuroD1*	R/I			
		*MafA*	R/I			
		*Nkx* *6.1*	R/I			
MafB	Musculoaponeurotic Fibrosarcoma Oncogene Homolog B	*GAST*	R	De	N	[168,169,170,171]
		*SST*	R			
		*P2ry1*	I	Tra	N	
		*Adra2A*	I			
		*Tph1*	I	Neo	P	
		*Ins*	I			
		*ChrnA3*, *ChrnA4*, *ChrnB4*	I	Pro	P	
		*Robo1*, *Robo2*, *Nrp1*, *Nrp2*	I			
Hes1	Hairy and Enhancer of Split-1	*Ngn3*	R	De	P	[172,173,174,175]
		*NeuroD*	R	Tra	P	
		*Ptf1a*	R	Neo	N	
		*p27Kip1 and p57Kip2*	R	Pro	P	
HNF4 A	Hepatocyte Nuclear Factor 4 A	*Aldolase B*	I	De	N	[176,177]
		*Glut2*	I			
		*Gck*	I	Tra	N	
		*L-pyruvate kinase*	I			
		*Kir6.2*	I	Neo	P	
		*Ins*	I			
		*Pdx1*	I	Pro	P	
		*Pax4*	I			
		*Ngn3*	I			
		*Ki67*	I			
NeuroD1	Neurogenic Differentiation 1	*Ins*	I	De	N	[178,179,180]
		*Gck*	I			
		*Abcc8/Sur1*, *Kcnj11*	I			
		*Cacna1a*	I	Tra	N	
		*Mafa*	I			
		*Nkx6-1*	I			
		*Pdx1*	I	Neo	P	
		*Nkx2-2*	I			
		*Insm1*	I			
		*Isl1*	I	Pro	P	
		*Pax6*	I			
		*Rfx6*	I			
		*Cdh4*	R			
		*Ocln*	R			
		*Mecom*	R			
		*ldha*	R			
*Nkx* *6.1*	NK6 homeobox 1	*Glut2*	I	De	N	[130,181,182,183,184]
		*G6pc2*	I			
		*Arx*	R	Tra	N	
		*Pdx1*	I			
		*MafA*	I	Neo	P	
		*Ero1lb*	I	Pro	P	
		*Slc30a8*	I			
		*Nr4a1 and Nr4a3*	I			
ISL1	ISL LIM homeobox 1	*MafA*	I	De	N	[185,186,187,188]
		*Pdx1*	I			
		*Glut2*	I			
		*Ins*	I			
		*NeuroD1*	I	Tra	N	
		*Pax6*	I			
		*MafB*,	I			
		*Nkx6.2*	I			
		*Insm1*	I	Neo	P	
		*Sox9*	I			
		*Arx*	I			
		*Foxa2*	I			
		*Nkx6.1*	I	Pro	P	
		*GCG*	I			
		*SST*	I			
		*G6pc2*	I			

**Table 3:** LncRNAs involved in β-cell dedifferentiation, transdifferentiation, neogenesis, or proliferation (De/Tra/Pro/Neo).

LncRNAs	Direct and Indirect Targets	De/Tra/Pro/Neo	Type of Effect	References
*Gm16308*		Pro	P	[243]
*DANCR*	*miR-33a-5p*	Pro	P	[218,244]
*H19*	*miRNA let-7- miRNA-29a*	Pro	P	[245,246,247]
*TUNAR*	*EZH2*	Pro	P	[248]
*Meg3*	*EZH2*, *Pdx1*, *MafA*	De	N	[249,250]
*βFaar*	*miR-138-5p*	De	N	[251]
*PLUTO*	*Pdx1*	De	N	[124]
*HILNC25*	*Glis3*	De	N	[252]
*Gm10451*	*miR-338-3p*	De	N	[253]
*810019D21Rik (RIOT)*	*Nkx6.1*	De	N	[254]
*p3134*	*Pdx1*	De	N	[124,255]
*linc-p21*	*miR-766-3p*	Pro	N	[256]
*HOTAIR*	*Pdx1*	De	N	[124]
*LncCplx2*	*EZH2*	Pro	N	[257]
*MALAT1*	*Pdx1*	De	P	[258]
*MIR503HG*	*CtBP1*	Neo	N	[175]
*GAS5*	*miR-29a-3p*, *miR-96-3p*, *miR-208a-3p*	De	N	[259,260]
		Pro	P	
*LINC01018*	*miR-499a-5p*	De	P	[261]
		Pro	N	

## Data Availability

Not applicable.
